# How old are you? A systematic review investigating the relationship between age and mandibular third molar maturity

**DOI:** 10.1371/journal.pone.0285252

**Published:** 2023-05-18

**Authors:** Gunilla Klingberg, Daniel Benchimol, Henrik Berlin, Johan Bring, Carl Gornitzki, Jenny Odeberg, Sofia Tranæus, Svante Twetman, Emma Wernersson, Pernilla Östlund, Helena Domeij

**Affiliations:** 1 Faculty of Odontology, Department of Pediatric Dentistry, Malmö University, Malmö, Sweden; 2 Department of Dental Medicine, Division of Oral Diagnostics and Rehabilitation, Karolinska Institutet, Huddinge, Sweden; 3 Statisticon AB, Uppsala, Sweden; 4 Swedish Agency for Health Technology Assessment and Assessment of Social Services, Stockholm, Sweden; 5 Faculty of Odontology, Health Technology Assessment–Odontology, Malmö University, Malmö, Sweden; 6 Faculty of Health and Medical Sciences, Department of Odontology, University of Copenhagen, Copenhagen, Denmark; Universidade Federal Fluminense, BRAZIL

## Abstract

**Introduction and objective:**

Radiographic evaluation of the maturity of mandibular third molars is a common method used for age estimation of adolescents and young adults. The aim of this systematic review was to examine the scientific base for the relationship between a fully matured mandibular third molar based on Demirjian’s method and chronological age, in order to assess whether an individual is above or below the age of 18 years.

**Methods:**

The literature search was conducted in six databases until February 2022 for studies reporting data evaluating the tooth maturity using Demirjian´s method (specifically stage H) within populations ranging from 8 to 30 years (chronological age). Two reviewers screened the titles and abstracts identified through the search strategy independently. All studies of potential relevance according to the inclusion criteria were obtained in full text, after which they were assessed for inclusion by two independent reviewers. Any disagreement was resolved by a discussion. Two reviewers independently evaluated the risk of bias using the assessment tool QUADAS-2 and extracted the data from the studies with low or moderate risk of bias. Logistic regression was used to estimate the relationship between chronological age and proportion of subjects with a fully matured mandibular third molar (Demirjian´s tooth stage H).

**Results:**

A total of 15 studies with low or moderate risk of bias were included in the review. The studies were conducted in 13 countries and the chronological age of the investigated participants ranged from 3 to 27 years and the number of participants ranged between 208 and 5,769. Ten of the studies presented the results as mean age per Demirjian´s tooth stage H, but only five studies showed the distribution of developmental stages according to validated age. The proportion of subjects with a mandibular tooth in Demirjian´s tooth stage H at 18 years ranged from 0% to 22% among males and 0 to 16% in females. Since the studies were too heterogenous to perform a meta-analysis or a meaningful narrative review, we decided to refrain from a GRADE assessment.

**Conclusion:**

The identified literature does not provide scientific evidence for the relationship between Demirjian´s stage H of a mandibular third molar and chronologic age in order to assess if an individual is under or above the age of 18 years.

## Introduction

One of the most common methods for evaluating chronologic age based on dental maturity was published by Demirjian et al. in 1973 [1]. This method defines eight developmental stages in tooth formation, labeled A through H. It was originally based on seven permanent teeth located on the left side of the lower jaw (the mandible). In a later study by Mincer et al. (1993) the development stages were applied on the eighth permanent tooth in each quadrant, also referred to as the third molar or wisdom tooth in the jaw [2]. While the rest of the permanent dentition usually is fully erupted and developed before the age of 15, the third molar appears later, and its root-part is the last to be completed. This is the reason why third molars are of interest in forensic age assessment of older adolescents and young adults.

The scoring of Demirjian’s stages of tooth formation is based on panoramic radiographs, a common imaging modality in dentomaxillofacial radiology since the 1950´s [3,4]. The images provide an overview of both the maxillary and mandibular dental arches, but the technology requires proper patient positioning for optimal image quality [5]. Demirjian’s method provides detailed criteria for all stages of tooth maturity (A-H) and the examining dentist classifies the developmental stage of a third molar in the mandible calculating the chronologic age using reference data sets, for example the one provided by Mincer et al [2]. The stages G and H are of special interest. The apices of the root, the part of the root where the blood-vessels and nerves enter the root, are still open in stage G, while in the following stage H “the apical end of the root canal is closed, and the periodontal membrane has a uniform width surrounding the root” [1]. Thus, stage H represents the final stage where the root of the tooth is fully formed and after which no further development can be recognized. Tooth formation is influenced by gender, ethnicity and genetic factors, but is generally regarded resistant from environmental stress. Nevertheless, factors such as nutritional state [6], episodes of high fever and certain drugs are known to affect the morphogenesis of teeth [7].

Many legal matters concern the age of 18 years. This age separates children from adults, which is important especially with regard to the Convention on the Right of the Child. Forensic age estimations of third molar development are often used to assess the probability of a person being 18 years or older. In connection with this, it has been debated if using stage H in Demirjian’s method is accurate and whether the vast majority of all individuals presenting root development stage H in mandibular third molars are 18 years or older [8,9]. A number of previous reviews have summarized studies investigating the relationship between mandibular third molar formation and age. Most of these did not investigate the Demirjian’s method [10], did not aim to be a systematic review [11,12], or did not present data assessing the relationship between chronological age and proportion of subjects with a fully matured mandibular third molar [9,13–20]. One systematic review was of potential interest, but the risk of bias, as assessed by the ROBIS tool [21], was high [8]. The objective of this paper was therefore to examine the relationship between a fully matured mandibular third molar, as determined by Demirjian’s method, and chronological age. The specific research question was “*Can Demirjian´s tooth stage H of a mandibular third molar be used to reliably assess if an individual has reached the age of 18 years*?”

## Methods

### Protocol and registration

This systematic review was conducted at The Swedish Agency for Health Technology Assessment and Assessment of Social Services (SBU). A preceding version was published as a governmental report in Swedish on commission of the Swedish government in October 2021 (report). The current systematic review is based on an updated literature search compared with the governmental report. SBU uses a peer-reviewed protocol for systematic reviews including pre-specified objectives, which is available from SBU upon request. The systematic review process follows the general concepts covered by PRISMA [22].

### Eligibility criteria

#### Inclusion criteria

A study was considered eligible if the reported data satisfied the following criteria for diagnostic studies:

- Population (P): A mandibular third molar in study-participants within the age of 8 to 30 years
- Index test (I): Demirjian´s method (stage H)
- Reference test (R): Chronological age according to records
- Outcome (O): Diagnostic accuracy reported as sensitivity/specificity or the correlation of age and stage of tooth development as determined by Demirjian´s method (stage H)

In addition, only cross-sectional studies, longitudinal studies or systematic reviews based on these study designs written in English, German or any of the Scandinavian languages, published in peer-reviewed journals were accepted.

#### Exclusion criteria

We excluded studies investigating tooth development in individuals with medical diagnoses affecting growth of the mandible, e.g., rheumatoid arthritis or malignant diseases treated with radiation or cytostatic agents, as well as studies using imaging techniques other than panoramic radiographs. We also excluded studies on deceased persons and studies including less than 50 individuals.

### Literature search

A systematic literature search was conducted by an information specialist in the databases Cochrane Library (Wiley), Embase (Elsevier), Medline (OvidSP), Epistomonikos, KSR Evidence and International HTA Database from inception until February 2022. In addition, references and citations from the included studies were retrieved from the database Scopus (Elsevier) and screened. The complete search strategies are listed in S1 Table. No ongoing systematic review investigating the question of interest was registered in Prospero (International prospective register of systematic reviews).

### Study selection

All titles and abstracts identified by the search strategy were screened independently by at least two authors, (HD, ST, ST, GK and HB), using Rayyan (https://www.rayyan.ai/). All studies of potential relevance according to the inclusion criteria were obtained in full text and the same pairs independently assessed them for inclusion. Any disagreements were resolved by discussions. Excluded full text publications are listed in S2 Table.

### Risk of bias assessment

Risk of bias assessment of included studies was performed independently by at least two reviewers (HD, ST, ST, GK and HB) using QUADAS-2, a tool with signaling questions included to help judge the risk of bias in diagnostic studies [23]. QUADAS-2 consists of four key domains where the risk of bias for the following parts are assessed: patient selection, index test, reference standard, and flow and timing. In this review, we added a fifth domain concerning the risk of bias due to insufficient data presentation (S3 Table). Before starting the assessments, all reviewers discussed the questions in each domain to achieve a common understanding as to how the risk of bias could affect the results in this specific research area. We rated each study as having low, moderate or high risk of bias.

### Data collection process

From each included study with low or moderate risk of bias, data was extracted and inserted into tables by one reviewer, followed by auditing of the data extraction by another reviewer. Any disagreements were resolved by discussion.

#### Data items

Information concerning study design, measurement of index test, reference test and outcome, as well as the population and setting, was extracted from each included study with low or moderate risk of bias.

### Method of analysis

The intention was to pool data from included studies. Due to heterogeneity between the included studies (described in the result section), such analyses were not undertaken. Instead, when possible, the data from each study was visualized as the association between age and the proportion of mandibular third molar in stage H. In addition, the relationship between age and proportion in stage H was estimated using a logistic regression model, where the dependent variable was the individual’s status (H or not H) and the independent variable was the individual’s age.

### Rating the certainty of the evidence

The intention was to rate the certainty of the evidence in the estimated pooled prevalence using GRADE (grading of recommendations assessment, development and evaluation, http://www.gradeworkinggroup.org). However, since the studies were too heterogenous to perform a meta-analysis or a meaningful narrative review, we decided to refrain from a GRADE assessment.

## Results

**Fig 1:**
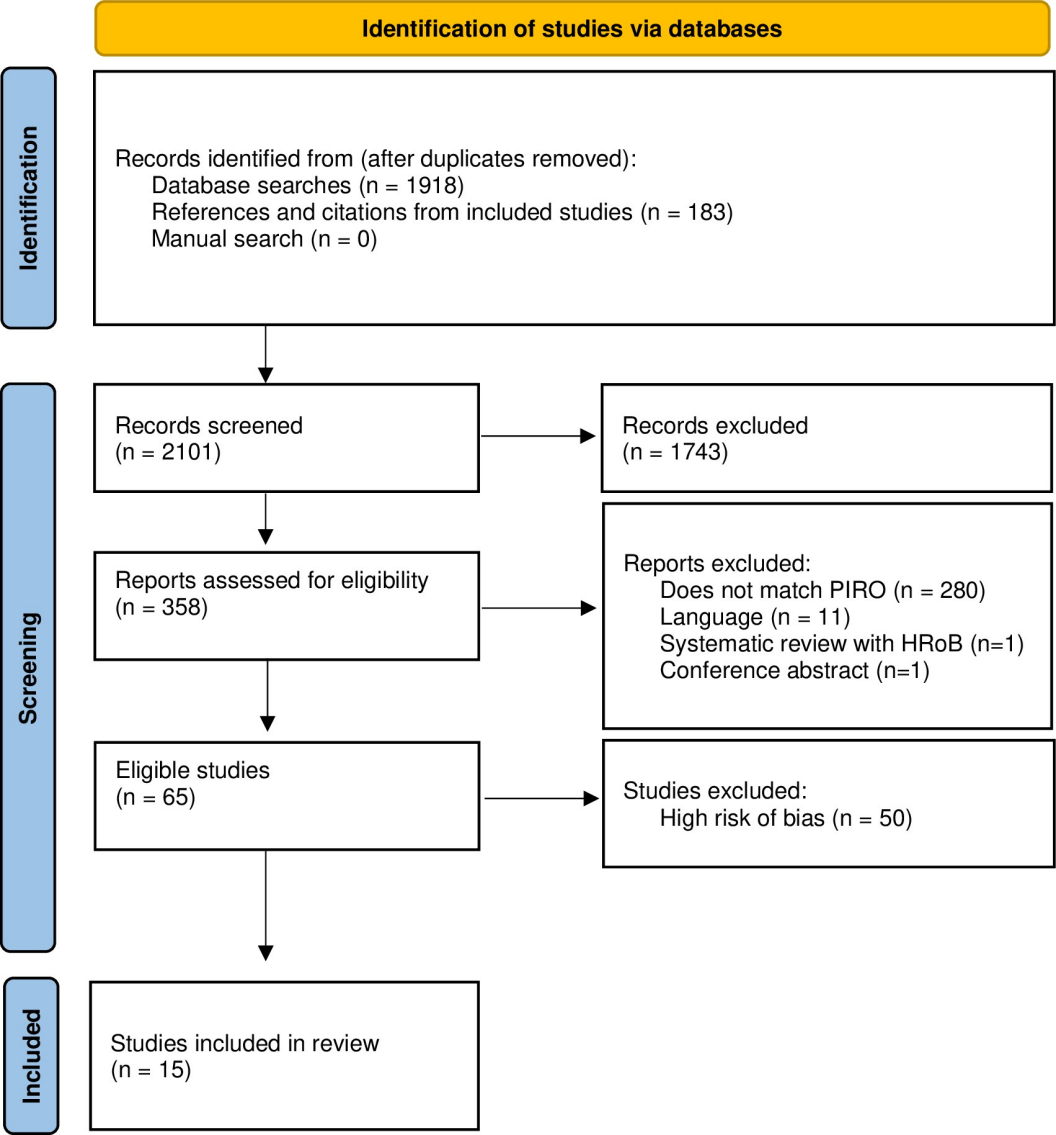
Flow diagram showing the literature review process.

A systematic literature search, first performed in March 2021 and updated in February 2022, resulted in 2,101 records, out of which 65 fulfilled the inclusion criteria (see flow chart in Fig 1). The main reason for exclusion were that the studies did not investigate Demirjian´s method or did not report data for at least one of the two lower mandibular third molars. The excluded studies are listed in S2 Table. After a risk of bias assessment of the relevant studies, we included 15 studies with a low or moderate risk of bias in this review [24–38]. We show the study characteristics in Table 1 and the risk of bias assessment in Table 2.

**Table 1:** Main characteristics of the included studies with low and moderate risk of bias.

First author, Year; Country (reference)	Sample type	Sample size female/male	Age, Years	Reference test	Reported outcome
Cantekin, 2012; Turkey [24]	Non-random	726/622	7–22	Records	Mean age by stage
Duangto, 2017; Thailand [25]	Random	990/877	8–23	Birth date	Tooth stage by age
Guo, 2014; China [27]	Random	1,024/510^A^	11–26	Birth date	Mean age by stage
Guo, 2015; China [26]	Non-random	1,661/1,551	5–25	Records	Mean age by stage
Hassan, 2021; Egypt [28]	Non-random	180/170	14–24	Records	Stage by age <18> yr
Kasper, 2009; USA [29]	Non-random	535/415	12–22	Birth certificate	Tooth stage by age
Lee, 2010; South Korea [30]	Random	1,057/1,030	3–23	Records	Mean age by stage
Li, 2012; China [31]	Random	1,089/989	5–23	Records	Tooth stage by age
Lopez, 2013; Brazil [32]	Non-random	379/280	15–23	Records	Mean age by stage
Memorando, 2020; Philippines [33]	Non-random	169/215	9–23	Birth date	Tooth stage by age
Mohammed, 2014; India [34]	Random	165/165	9–20	Records	Mean age by stage
Mwesigwa, 2019; Uganda [35]	Random	262/258	10–22	Birth certificate	Mean age by stage
Quispe Lizarbe, 2017; Peru [36]	Non-random	106/102	14–22	Records	Mean age by stage
Zandi, 2015; Iran [37]	Random	1,554/982	5–26	Records	Mean age by stage
Zeng, 2010; China [38]	Non-random	1,900/1,200	4–27	Records	Mean age by stage

All studies had a retrospective cross-sectional design and used the developmental stages of the mandibular third molars from panoramic radiographs according to Demirjian et al. [1] as index test. ^A^ non-impacted teeth.

**Table 2:** Quality assessment of the included primary studies with low or moderate risk of bias.

	Risk of bias					
Study	Patient selection	Index test	Reference standard	Flow and timing	Data	Total risk of bias
Cantekin 2012 [24]						**M **
Duangto 2017 [25]						**M **
Guo 2014 [27]						**M **
Guo 2015 [26]						**M **
Hassan 2021 [28]						**L **
Kasper 2009 [29]						**M **
Lee 2010 [30]						**L **
Li 2012 [31]						**L **
Lopez 2013 [32]						**M **
Memorando 2020 [33]						**M **
Mohammed 2014 [34]						**M **
Mwesigwa 2019 [35]						**M **
Quispe Lizarbe 2017 [36]						**M **
Zandi 2015 [37]						**M **
Zeng 2010 [38]						**M **

Risk of bias in the five different domains showing low risk of bias in green, unclear risk of bias in yellow and high risk of bias in red. Total risk of bias is either moderate (M) or low (L).

The 50 studies with high risk of bias had serious methodological inconsistencies, mainly concerning the index test, reference test, patient selection and data presentation (S4 Table).

**Fig 2:**
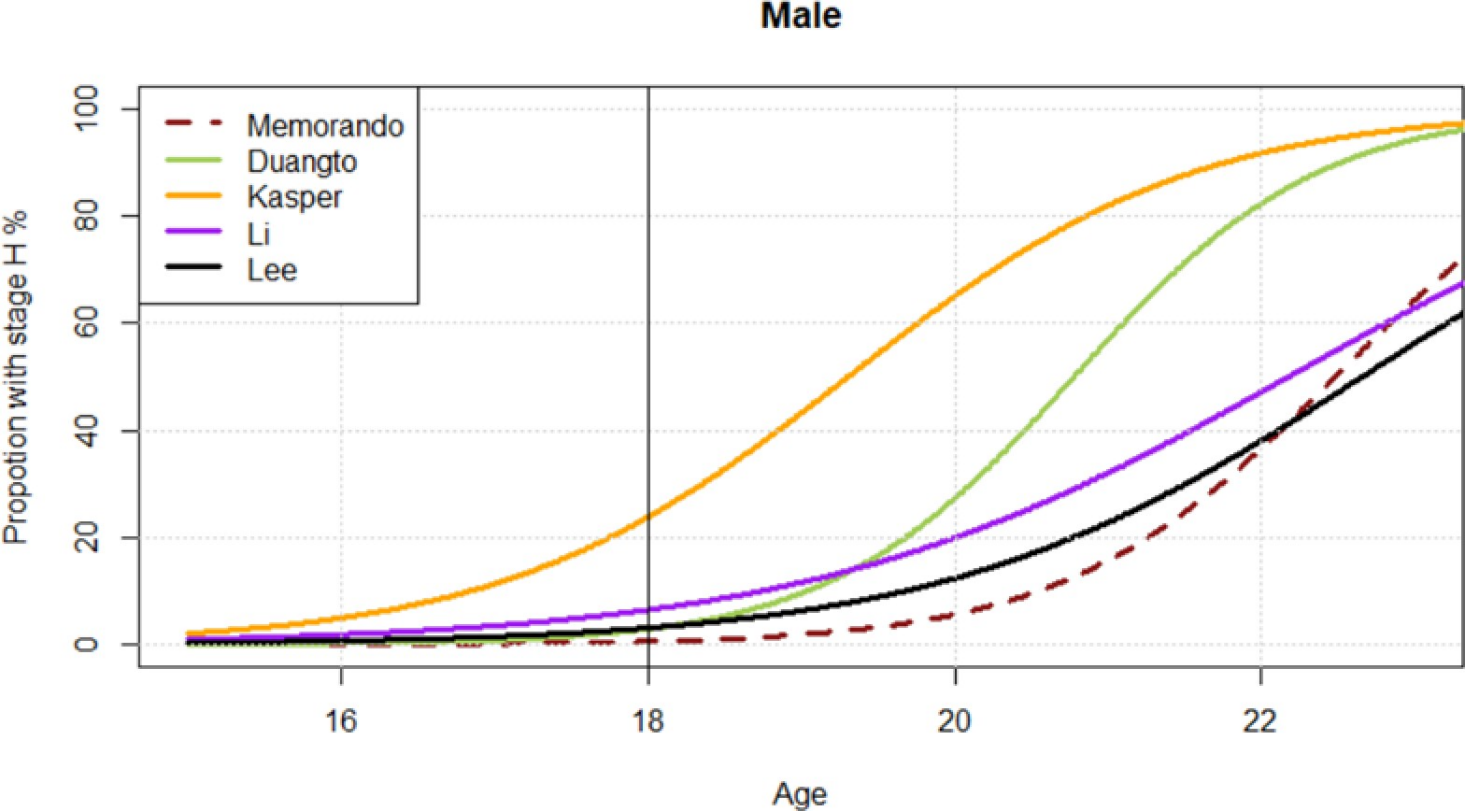
Estimated proportions of mandibular third molar in stage H for males according to age. *Memorando et al. [33] includes both females and males.

**Fig 3:**
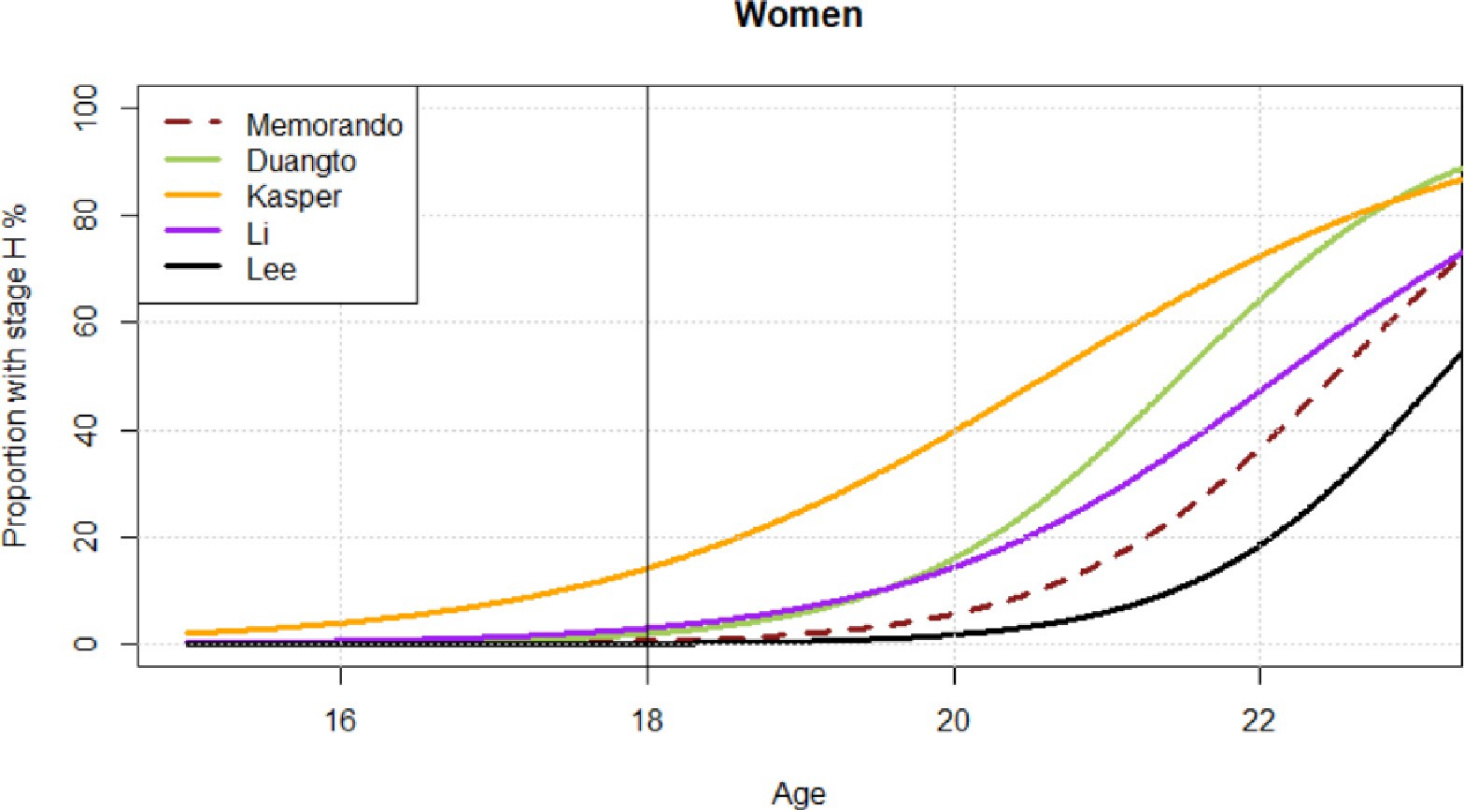
Estimated proportions of mandibular third molar in stage H for females according to age. *Memorando et al. [33] includes both females and males.

The studies with low or moderate risk of bias [24–38] were conducted in thirteen countries and investigated children and young adults between the age of 3 and 27 years (Table 1). The number of investigated individuals varied between 208 and 5,769, but in number of studies, the age distribution was uneven. The most common result presentation was mean age per Demirjian´s tooth stage (Table 1), which was not useful for our research question. In order to estimate the association between chronological age and a matured mandibular third molar (Demirjian’s stage H) on individual level, data is required as developmental stage in relation to a validated age. Only five studies described their data in such a way [25,29,31,33], or in a way that allowed a recalculation of the data [30]. These five studies described 7,366 subjects between 3 and 23 years of age from populations in Thailand [25], USA (Hispanic Texas) [29], South Korea [30], China [31] and the Philippines [33]. In Figs 2 and 3, we show the proportions of males and females with a fully developed mandibular third molar in relation to the validated chronological age. The proportion of individuals with a mandibular third molar in stage H at 18 years ranged from 0 to 22% among males and 0 to 16% among females.

**Fig 4:**
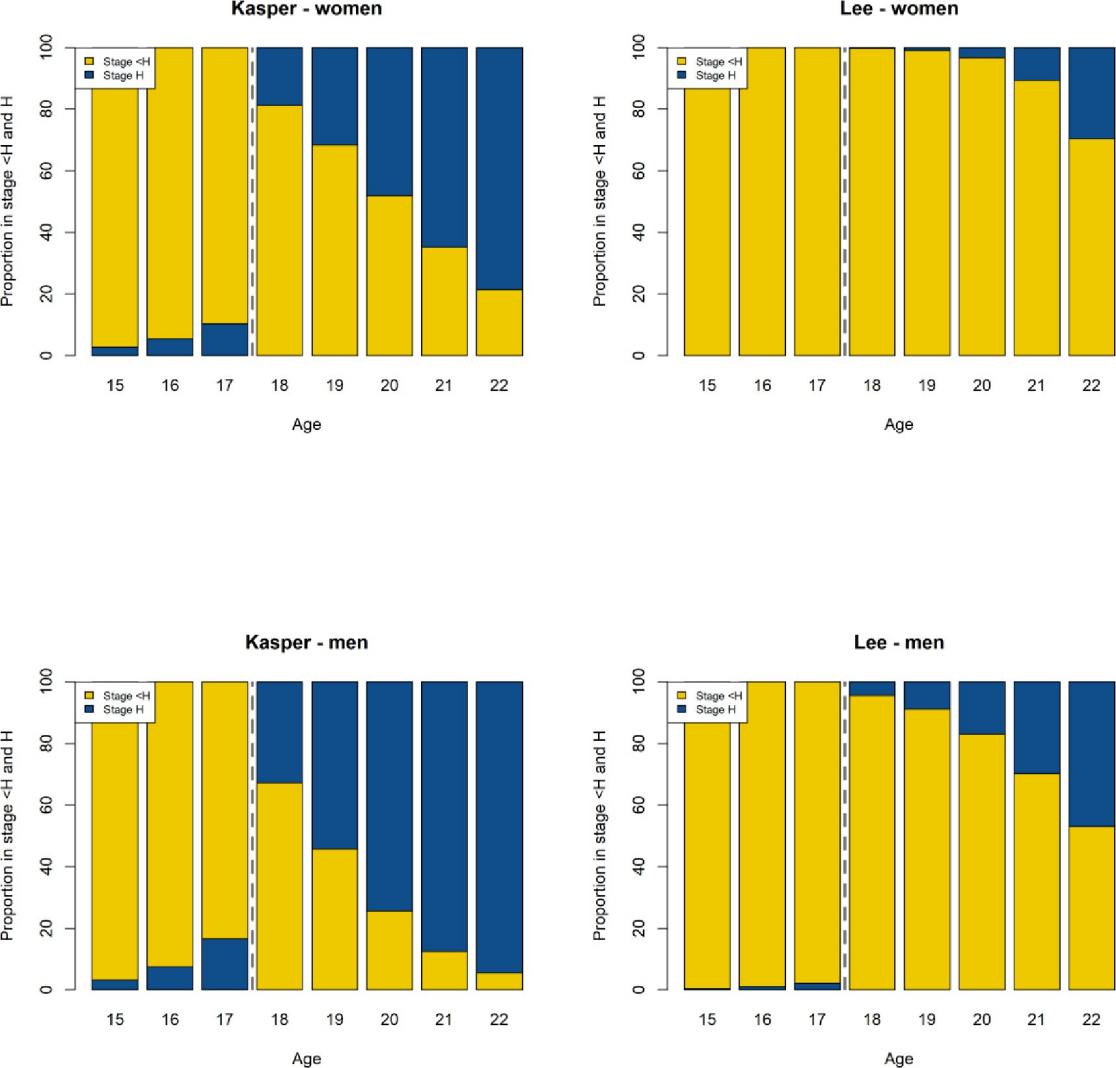
The proportion of mandibular third molar in males and females with stages <H and H in different age groups. Data from Kasper et al., 2009 [29] and Lee et al., 2010 [30].

The risk of being classified as ≥18 years of age when younger, or vice versa, is presented in Fig 4, and is based on data from the two most contrasting studies [29,30]. With a fully developed mandibular third molar (stage H), there was a lower risk of being classified as under 18 years for both sexes. Conversely, the risk was higher for an individual over 18 years to be regarded as younger than 18 years.

### Certainty of evidence

The intention was to rate the certainty of the evidence in an estimated pooled prevalence using GRADE (grading of recommendations assessment, development and evaluation, http://www.gradeworkinggroup.org). However, as explained above, the data from included studies was not pooled and a synthesis without meta-analysis (SWiM) was neither applicable. Hence, the certainty of evidence was regarded as very low, and the effect could therefore not be determined.

## Discussion

This systematic review could not determine the evidence for the agreement between Demirjian’s development stage H of the mandibular third molar and chronological age. The studies that were identified in this review do not show that stage H of mandibular third molars can be used to assess if an individual is under or above the age of 18 years in a reliable way.

### From a statistical point of view

When the age is unclear one could predict age or an age-range for an individual, based on matured mandibular third molar. For statistical purpose the terms positive or negative predictive value (PPV or NPV) would therefore be considered. The problem in this case is that PPV and NPV depend on the age-distribution of those that are tested. In a study where the majority of investigated subjects are children, the PPV for the Demirjian´s method is low whereas in a study with older individuals the PPV is high. If an estimated age prevalence is applied (as a solution) a situation of circular reasoning occurs, a logic that proves a conclusion with itself. In that case, statistical measurements such as PPV, NPV as well as sensitivity and specificity calculated on comparison of such groups will lead to incorrect conclusions. It will also result in incorrect calculations of incidence. Since the results depends on the age of the studied individuals, the likelihood of determining correct age by investigating third molar maturity may not be valuable. It is however possible to calculate the risk for misclassification for each age-group, as we have shown for each of the included study.

### From a clinical point of view

It is important to recognize that individuals mature differently, which directly affects the development of teeth including the third molars. This is especially true across populations as illustrated in Fig 4. The third molar development was significantly faster in the American-Hispanic population described by Kasper et al. [29] compared with the Korean population reported by Lee et al. [30]. The assessment based on the maturity of the mandibular third molars in panoramic radiographs is also inevitably dependent on the actual presence of one of the two mandibular third molars. These teeth have been described as characterized by a higher proportion of congenital absence than other teeth [39]. Other reasons for missing mandibular third molars can be due to extractions induced by pericoronitis, root resorption, cysts, caries and periodontitis.

### From a technical point of view

Other drawbacks to be aware of concerns imaging technique and positioning of the patient when taking radiographs. All the studies with low or medium risk bias that were identified in this review declared that they excluded patients if there was distortion of the depicted third molar or other quality problems like image deformity or obvious dental pathology. But there can also be more subtle issues that are not always possible to detect. The projection-based visualization in panoramic imaging of the apical part of the mandibular third molars can be limited due to the angulation of the teeth in relation to the beam direction making the apical part inaccessible. As for most interpretations the image quality is crucial and can, if inadequate, reduce the information considerably.

In the future new methods such as machine learning might be applicable in age assessment.

Machine learning enables automated detection of findings within radiographs based on computer algorithms with the ability to learn from data sets without being programmed with explicit rules [40]. Recent research indicates that machine learning has promising potential in age classification based on teeth when compared to the traditional manual Demirjian method [41]. This systematic review only included studies where the radiographs were analyzed by dentists, and machine learning techniques were not assessed.

### From an ethical point of view

Estimating age of an individual based on mandibular third molar maturity raises ethical questions as previously described by for example Thevissen et al. [42], and De Micco et al. [43]. An assessment of ethical aspects in systematic reviews is preferably based on the framework described by Heintz et al. [44]. Already in 2018 Malmqvist et al. [45] published an article on ethical aspects of medical ages assessment using the framework and identified several ethical issues. The present systematic review focusing on dental age assessment based on mandibular third molar maturity agrees with their results and conclusion. The main ethical issues identified in this systematic review concern compatibility the following items identified in the framework: knowledge gaps (if there is insufficient scientific evidence to support the effect of the method), autonomy (are the patients able to consent to the intervention?), equality and justice (if there is a risk that access to the method violates the principle of human value or current legislation), special interests (if there are special interests, which can influence implementation of the method), and long terms consequences (if the application of the intervention have ethical consequences in the long term?). Thus, ethical standards are of interest and should be incorporated in all decision making concerning the possible use of age assessments based on mandibular third molar maturity.

### Future studies

For research questions with other purposes than dichotomous age assessment around 18 years of age, the present systematic review can be of help when planning the outline of the project. The studies with high risk of bias in the present systematic review had common problems. One main issue concern aspects of study design. Future studies should ensure and provide information on training and calibration of observers, having more than one dentist responsible for assessments and calculate intra- and interobserver reliability. Also blinding of radiographs to ensure that the observer is not familiar with identity of the patient is essential. All these aspects should also be described in the publications. Patients were often included retrospectively, and the researchers’ gathered data based on cases available at a dental clinic or dental hospital. This approach is suboptimal as patients seeking dental care may not mirror the background population. On the other hand, from an ethical standpoint, it is not possible to have a population-based random inclusion of individuals when radiation is involved. The retrospective design also implies problems to secure the index test, to assure the true identity (and thereby age) of the person behind the radiographs. The process of assuring identity was often poorly described in the included studies. For future studies, a prospective inclusion of patients with a relevant age-distribution as well as assuring large number of individuals in each age group is advocated. Finally, results presented with distribution of developmental stages according to validated age (in complement to the age-distribution per development stages) are essential to be able to calculate many important statistical measures.

## Conclusion

The identified literature does not provide scientific evidence for a stable relationship between Demirjian´s stage H of a mandibular third molar and chronologic age in order to assess if an individual is under or above the age of 18 years. This relationship varies considerably between different populations.

## Supporting information

S1 Table Search strategy. (DOCX)

S2 Table Excluded studies. (DOCX)

S3 Table Quality assessment tool. (DOCX)

S4 Table Quality assessment of studies with high risk of bias. (DOCX)
