# Supplementary material for: How old are you? A systematic review investigating the relationship between age and mandibular third molar maturity
Source: PLoS One. 2023 May 18;18(5):e0285252. doi: 10.1371/journal.pone.0285252 (PMC10194975; doi:10.1371/journal.pone.0285252)
Supplement: S1 Table — (DOCX) [file pone.0285252.s001.docx]

**S1. Search strategy**

**Cochrane Library**

| Search terms | |
| --- | --- |
|  | ((age OR matur* OR year*) NEAR/5 (assess* OR chronologic* OR determ* OR estimat* OR evaluat* OR examin* OR forensic OR ident* OR legal OR measur* OR predict* OR verif*)):ti,ab,kw |
|  | (forensic NEAR/3 (dent* OR odont*)):ti,ab,kw |
|  | *1 OR 2* |
|  | ("3rd molar*" OR "wisdom teeth*" OR "wisdom tooth*"):ti,ab,kw |
|  | (third NEAR/3 molar*):ti,ab,kw |
|  | m3:ti,ab,kw AND (dent* or odont*):ti,ab,kw |
|  | *4 OR 5 OR 6* |
|  | *3 AND 7* |

**:ti,ab,kw** = Title, abstract or keyword; ***** = Truncation; " " = Citation Marks; searches for an exact phrase; **CDSR** = Cochrane Database of Systematic Review; **Cochrane Protocols** = Protocols of systematic reviews registered in Cochrane Library; **CENTRAL** = Cochrane Central Register of Controlled Trials, “trials”

Embase

| Search terms | |
| --- | --- |
|  | 'age determination'/exp |
|  | 'forensic odontology'/de |
|  | ((age OR matur* OR year*) NEAR/5 (assess* OR chronologic* OR determ* OR estimat* OR evaluat* OR examin* OR forensic OR ident* OR legal OR measur* OR predict* OR verif*)):ti,ab,kw |
|  | (forensic NEAR/3 (dent* OR odont*)):ti,ab,kw |
|  | *1 OR 2 OR 3 OR 4* |
|  | 'third molar'/exp |
|  | '3rd molar*':ti,ab,kw OR 'wisdom teeth*':ti,ab,kw OR 'wisdom tooth*':ti,ab,kw |
|  | (third NEAR/3 molar*):ti,ab,kw |
|  | m3:ti,ab,kw AND ('dentition'/exp OR 'dentistry'/exp OR dent*:ti,ab,kw OR odont*:ti,ab,kw) |
|  | *6 OR 7 OR 8 OR 9* |
|  | demirjian*:ti,ab,kw |
|  | *5 AND 10* |
|  | *11 OR 12* |

**de** = Term from the EMTREE controlled vocabulary; **/exp** = Includes terms found below this term in the EMTREE hierarchy; **:ti,ab,kw** = Title, abstract or keyword; ***** = Truncation; **' '** = Citation Marks, searches for an exact phrase; **NEAR/n** = Requests terms that are within 'n' words of each other in either direction

*OBS!
Sökdokumentationen räknas som arbetsmaterial och får inte spridas utanför projektgruppen innan rapporten publiceras. Om sökstrategin i sin helhet används i andra sammanhang (t.e.x vid publicerande av artikel) bör man hänvisa till den publicerade sökdokumentationen på* [*www.sbu.se*](http://www.sbu.se)

*OBS!
Sökdokumentationen räknas som arbetsmaterial och får inte spridas utanför projektgruppen innan rapporten publiceras. Om sökstrategin i sin helhet används i andra sammanhang (t.e.x vid publicerande av artikel) bör man hänvisa till den publicerade sökdokumentationen på* [*www.sbu.se*](http://www.sbu.se)

*OBS!*

*Sökdokumentationen räknas som arbetsmaterial och får inte spridas utanför projektgruppen innan rapporten publiceras. Om sökstrategin i sin helhet används i andra sammanhang (t.e.x vid publicerande av artikel) bör man hänvisa till den publicerade sökdokumentationen på* [*www.sbu.se*](http://www.sbu.se)

Medline

| Search terms | |
| --- | --- |
|  | Age Determination by Teeth/ |
|  | Age Factors/ |
|  | Forensic Dentistry/ |
|  | ((age or matur* or year*) adj5 (assess* or chronologic* or determ* or estimat* or evaluat* or examin* or forensic or ident* or legal or measur* or predict* or verif*)).ti,ab,kf. |
|  | (forensic adj3 (dent* or odont*)).ti,ab,kf. |
|  | *1 OR 2 OR 3 OR 4 OR 5* |
|  | Molar, Third/ |
|  | (3rd molar* or wisdom teeth* or wisdom tooth*).ti,ab,kf. |
|  | (third adj3 molar*).ti,ab,kf. |
|  | m3.ti,ab,kf. and (exp Dentition/ or exp Dentistry/ or (dent* or odont*).ti,ab,kf.) |
|  | *7 OR 8 OR 9 OR 10* |
|  | demirjian*.ti,ab,kf. |
|  | *6 AND 11* |
|  | *12 OR 13* |

**ti.ab,kf.** = Title, abstract or Keyword Heading Word; **/** = Term from the Medline controlled vocabulary, but does not include terms (if existing) found below this term in the MeSH hierarchy; ***** = Truncation (if found at the end of a free text term); *adjN* = Positional operator that lets you retrieve records that contain your terms (in any order) within a specified number (n) of words of each other

Epistemonikos

| Search terms | |
| --- | --- |
|  | (title:((age OR matur* OR year*) AND (assess* OR chronologic* OR determ* OR estimat* OR evaluat* OR examin* OR forensic OR ident* OR legal OR measur* OR predict* OR verif*)) OR abstract:((age OR matur* OR year*) AND (assess* OR chronologic* OR determ* OR estimat* OR evaluat* OR examin* OR forensic OR ident* OR legal OR measur* OR predict* OR verif*))) AND (title:((third AND molar) OR ("3rd molar*" OR "wisdom teeth*" OR "wisdom tooth*")) OR abstract:((third AND molar) OR ("3rd molar*" OR "wisdom teeth*" OR "wisdom tooth*"))) OR (title:(demirjian*) OR abstract:(demirjian*)) |

***** = Truncation

KSR Evidence

| Search terms | |
| --- | --- |
|  | (age OR matur* OR year*) AND (assess* OR chronologic* OR determ* OR estimat* OR evaluat* OR examin* OR forensic OR ident* OR legal OR measur* OR predict* OR verif*) in All text |
|  | (third AND molar) OR ("3rd molar*" OR "wisdom teeth*" OR "wisdom tooth*") in All text |
|  | demirjian* in All text |
|  | *1 AND 2* |
|  | *3 OR 4* |

**All text =** All fields; ***** = Truncation; " " = Citation Marks; searches for an exact phrase

**International HTA Database**

| Search terms | |  |
| --- | --- | --- |
| **Age assessment** | | |
|  | (age OR matur* OR year*) AND (assess* OR chronologic* OR determ* OR estimat* OR evaluat* OR examin* OR forensic OR ident* OR legal OR measur* OR predict* OR verif*) in All |  |
| 1. **Third molar** | | |
|  | (third AND molar) OR ("3rd molar*" OR "wisdom teeth*" OR "wisdom tooth*") in All |  |
| 1. **Demirjian method** | | |
|  | demirjian* in All |  |
| 1. **Combined sets** | | |
|  | *1 AND 2* |  |
|  | *3 OR 4* |  |

**All =** All fields; ***** = Truncation; "**"** = Citation Marks; searches for an exact phrase
