# Supplementary material for: How old are you? A systematic review investigating the relationship between age and mandibular third molar maturity
Source: PLoS One. 2023 May 18;18(5):e0285252. doi: 10.1371/journal.pone.0285252 (PMC10194975; doi:10.1371/journal.pone.0285252)
Supplement: S2 Table — (DOCX) [file pone.0285252.s002.docx]

**S2. List of excluded studies**

| **Ref no.** | **Reference** | **Reason for exclusion (PIRO or other reason)** |
| --- | --- | --- |
|  | Abesi F, Haghanifar S, Sajadi P, Valizadeh A, Khafri S. Association between body mass index and dental development in 7-15-year-old children and adolescents in the city of Babol-Iran (2011). Journal of Babol University of Medical Sciences 2013;15:52-58. | Does not match PIRO |
|  | Abu Asab S, Noor SNFM, Khamis MF. The accuracy of demirjian method in dental age estimation of malay children. Singapore dental journal 2011;32:19-27. | Does not match PIRO |
|  | Acharya AB, Bhowmik B, Naikmasur VG. Accuracy of identifying juvenile/adult status from third molar development using prediction probabilities derived from logistic regression analysis. Journal of forensic sciences 2014;59:665-670. | Does not match PIRO |
|  | Acharya AB. Age estimation in Indians using Demirjian's 8-teeth method. Journal of forensic sciences 2011;56:124-127. | Does not match PIRO |
|  | Agrawal NK, Hackman L, Dahal S. Dental Age Assessment using Demirjian's Eight Teeth Method and Willems Method in a Tertiary Hospital. JNMA; journal of the Nepal Medical Association 2018;56:912-916. | Does not match PIRO |
|  | Aissaoui A, Salem NH, Mougou M, Maatouk F, Chadly A. Dental age assessment among Tunisian children using the Demirjian method. Journal of forensic dental sciences 2016;8:47-51. | Does not match PIRO |
|  | Akhil S, Joseph TI, Girish KL, Sathyan P. Accuracy of Demirjian's and Indian-specific formulae in age estimation using eight-teeth method in Kanyakumari population. Indian journal of dental research : official publication of Indian Society for Dental Research 2019;30:352-357. | Does not match PIRO |
|  | Akkaya N, Yilanci HO, Goksuluk D. Applicability of Demirjian's four methods and Willems method for age estimation in a sample of Turkish children. Legal medicine (Tokyo, Japan) 2015;17:355-359. | Does not match PIRO |
|  | Akkaya N, Yilanci HO, Boyacioglu H, Goksuluk D, Ozkan G. Accuracy of the use of radiographic visibility of root pulp in the mandibular third molar as a maturity marker at age thresholds of 18 and 21. International journal of legal medicine 2019;133:1507-1515. | Does not match PIRO |
|  | Akkaya N, Yilanci HÖ. Assessment of third molar maturity index for legal age threshold of 18 in a sample of Turkish individuals. Australian Journal of Forensic Sciences 2020. | Does not match PIRO |
|  | Al Balushi S, Thomson WM, Al-Harthi L. Dental age estimation of Omani children using Demirjian's method. The Saudi dental journal 2018;30:208-213. | Does not match PIRO |
|  | Al Qattan F, Alzoubi EE, Lucas V, Roberts G, McDonald F, Camilleri S. Root Pulp Visibility as a mandibular maturity marker at the 18-year threshold in the Maltese population. International journal of legal medicine 2020;134:363-368. | Does not match PIRO |
|  | Alassiry A, Alshomrani K, Al Hasi S, Albasri A, Alkhathami SS, Althobaiti MA. Dental age assessment of 3-15-year-old Saudi children and adolescents using Demirjian's method-A radiographic study. Clinical and experimental dental research 2019;5:336-342. | Does not match PIRO |
|  | Albernaz Neves J, Antunes-Ferreira N, Machado V, Botelho J, Proenca L, Quintas A, et al. Validation of the Third Molar Maturation Index (I3M) to assess the legal adult age in the Portuguese population. Scientific reports 2020;10:18466. | Does not match PIRO |
|  | Al-Emran S. Dental age assessment of 8.5 to 17-Year-old Saudi children using Demirjian's method. The journal of contemporary dental practice 2008;9:64-71. | Does not match PIRO |
|  | AlQahtani S, Kawthar A, AlAraik A, AlShalan A. Third molar cut-off value in assessing the legal age 18 in Saudi population. Forensic science international 2017;272:64-67. | Does not match PIRO |
|  | Alsaffar H, Elshehawi W, Roberts G, Lucas V, McDonald F, Camilleri S. Dental age estimation of children and adolescents: Validation of the Maltese Reference Data Set. Journal of forensic and legal medicine 2017;45:29-31. | Does not match PIRO |
|  | Altalie S, Thevissen P, Fieuws S, Willems G. Optimal dental age estimation practice in United Arab Emirates' children. Journal of forensic sciences 2014;59:383-385. | Does not match PIRO |
|  | Altan H, Altan A, Sozer OA. Dental age estimation in southern Turkish children: Comparison of Demirjian and Willems methods. Iranian Journal of Pediatrics 2017;27. | Does not match PIRO |
|  | Altunsoy M, Nur BG, Akkemik O, Ok E, Evcil MS. Applicability of the Demirjian method for dental age estimation in western Turkish children. Acta odontologica Scandinavica 2015;73:121-125. | Does not match PIRO |
|  | Ambarkova V, Galic I, Vodanovic M, Biocina-Lukenda D, Brkic H. Dental age estimation using Demirjian and Willems methods: cross sectional study on children from the Former Yugoslav Republic of Macedonia. Forensic science international 2014;234:187.e181-187. | Does not match PIRO |
|  | Angelakopoulos N, De Luca S, Velandia Palacio LA, Coccia E, Ferrante L, Cameriere R. Third molar maturity index (I3M) for assessing age of majority: study of a black South African sample. International journal of legal medicine 2018;132:1457-1464. | Does not match PIRO |
|  | Antunovic M, Mihajlovic KZ, Nedeljkovic N, Lazic E, Galic I. Third molars in assessing legal adulthood on montenegrin population. Acta Stomatologica Croatica 2016;50:188. | Conference abstract |
|  | Antunovic M, Galic I, Zelic K, Nedeljkovic N, Lazic E, Djuric M, et al. The third molars for indicating legal adult age in Montenegro. Legal medicine (Tokyo, Japan) 2018;33:55-61. | Does not match PIRO |
|  | Apaydin BK, Yasar F. Accuracy of the demirjian, willems and cameriere methods of estimating dental age on turkish children. Nigerian journal of clinical practice 2018;21:257-263. | Does not match PIRO |
|  | Arge S, Boldsen JL, Wenzel A, Holmstrup P, Jensen ND, Lynnerup N. Third molar development in a contemporary Danish 13-25year old population. Forensic science international 2018;289:12-17. | Does not match PIRO |
|  | Arge S, Wenzel A, Holmstrup P, Jensen ND, Lynnerup N, Boldsen JL. Transition analysis applied to third molar development in a Danish population. Forensic science international 2020;308:110145. | Does not match PIRO |
|  | Arthanari A, Doggalli N, Vidhya A, Rudraswamy S. Age estimation from second & third molar by modified gleiser and hunt method: A retrospective study. Indian Journal of Forensic Medicine and Toxicology 2020;14:1-8. | Does not match PIRO |
|  | Artis O, Coudane H, Artis JP. Estimation of the age from the stage of development of wisdom teeth, in a caucasian population of the north-east of France. Journal de Medecine Legale Droit Medical 2007;50:399-407. | Language |
|  | Asif MK, Ibrahim N, Al-Amery SM, John J, Nambiar P. Juvenile versus adult: A new approach for age estimation from 3-dimensional analyses of the mandibular third molar apices. Journal of Forensic Radiology and Imaging 2019;19. | Does not match PIRO |
|  | Asif MK, Nambiar P, Ibrahim N, Al-Amery SM, Khan IM. Three-dimensional image analysis of developing mandibular third molars apices for age estimation: A study using CBCT data enhanced with Mimics & 3-Matics software. Legal medicine (Tokyo, Japan) 2019;39:9-14. | Does not match PIRO |
|  | Baghdadi ZD, Pani SC. Accuracy of population-specific Demirjian curves in the estimation of dental age of Saudi children. International journal of paediatric dentistry 2012;22:125-131. | Does not match PIRO |
|  | Baghdadi ZD. Dental maturity in saudi children using the demirjian method: a comparative study and new prediction models. ISRN dentistry 2013;2013:390314. | Does not match PIRO |
|  | Baghdadi ZD. Dental maturity of Saudi children: Role of ethnicity in age determination. Imaging science in dentistry 2013;43:267-272. | Does not match PIRO |
|  | Baghdadi ZD. Testing international dental maturation scoring system and population-specific Demirjian versions on Saudi sub-population. Journal of clinical and experimental dentistry 2014;6:e138-144. | Does not match PIRO |
|  | Bagherpour A, Anbiaee N, Partovi P, Golestani S, Afzalinasab S. Dental age assessment of young Iranian adults using third molars: A multivariate regression study. Journal of forensic and legal medicine 2012;19:407-412. | Does not match PIRO |
|  | Balla SB, Galic I, P K, Vanin S, De Luca S, Cameriere R. Validation of third molar maturity index (I3M) for discrimination of juvenile/adult status in South Indian population. Journal of forensic and legal medicine 2017;49:2-7. | Does not match PIRO |
|  | Balla SB, Banda TR, Galic I, N NM, Naishadham PP. Validation of Cameriere's third molar maturity index alone and in combination with apical maturity of permanent mandibular second molar for indicating legal age of 14 years in a sample of South Indian children. Forensic science international 2019;297:243-248. | Does not match PIRO |
|  | Balla SB, Chinni SS, Galic I, Alwala AM, Machani P, Cameriere R. A cut-off value of third molar maturity index for indicating a minimum age of criminal responsibility: Older or younger than 16 years? Journal of forensic and legal medicine 2019;65:108-112. | Does not match PIRO |
|  | Balla SB, Lingam S, Kotra A, P HR, P K, N NM, et al. New regression models for dental age estimation in children using third molar maturity index: A preliminary analysis testing its usefulness as reliable age marker. Legal medicine (Tokyo, Japan) 2019;39:35-40. | Does not match PIRO |
|  | Balla SB, Ankisetti SA, Bushra A, Bolloju VB, Mir Mujahed A, Kanaparthi A, et al. Preliminary analysis testing the accuracy of radiographic visibility of root pulp in the mandibular first molars as a maturity marker at age threshold of 18 years. International journal of legal medicine 2020;134:769-774. | Does not match PIRO |
|  | Balla SB, Kollata VRS, Bathala V, Ganapathy SK, Ch G, Vedula P, et al. Evaluation of the effect of impaction on the mineralization of mandibular third molars and forensic age estimation in a sample of south Indian children. Int J Legal Med. 2021 Oct 15. doi: 10.1007/s00414-021-02713-w | Does not match PIRO |
|  | Bassed RB, Briggs C, Drummer OH. Age estimation and the developing third molar tooth: an analysis of an Australian population using computed tomography. Journal of forensic sciences 2011;56:1185-1191. | Does not match PIRO |
|  | Bassed RB, Briggs C, Drummer OH. Age estimation using CT imaging of the third molar tooth, the medial clavicular epiphysis, and the spheno-occipital synchondrosis: a multifactorial approach. Forensic science international 2011;212:273.e271-275. | Does not match PIRO |
|  | Baumann P, Widek T, Merkens H, Boldt J, Petrovic A, Urschler M, et al. Dental age estimation of living persons: Comparison of MRI with OPG. Forensic science international 2015;253:76-80. | Does not match PIRO |
|  | Bhat VJ, Kamath G. Age estimation from the root development of mandibular third molars. Medico-Legal Update 2004;4:127-130. | Does not match PIRO |
|  | Bhowmik B, Acharya AB, Naikmasur VG. The usefulness of Belgian formulae in third molar-based age assessment of Indians. Forensic science international 2013;226:300.e301-305. | Does not match PIRO |
|  | Bijjaragi SC, Sangle VA, Saraswathi FK, Patil VS, Ashwini Rani SR, Bapure SK. Age estimation by modified Demirjian's method (2004) and its applicability in Tibetan young adults: A digital panoramic study. Journal of oral and maxillofacial pathology : JOMFP 2015;19:100-105. | Does not match PIRO |
|  | Birchler FA, Kiliaridis S, Combescure C, Vazquez L. Dental age assessment on panoramic radiographs in a Swiss population: a validation study of two prediction models. Dento maxillo facial radiology 2016;45:20150137. | Does not match PIRO |
|  | Birchler FA, Kiliaridis S, Combescure C, Julku J, Pirttiniemi PM, Vazquez L. Dental age assessment on panoramic radiographs: Comparison between two generations of young Finnish subjects. The Journal of international medical research 2019;47:311-324. | Does not match PIRO |
|  | Bittencourt MV, Cericato G, Franco A, Girao R, Lima APB, Paranhos L. Accuracy of dental development for estimating the pubertal growth spurt in comparison to skeletal development: a systematic review and meta-analysis. Dento maxillo facial radiology 2018;47:20170362. | Does not match PIRO |
|  | Blankenship JA, Mincer HH, Anderson KM, Woods MA, Burton EL. Third molar development in the estimation of chronologic age in american blacks as compared with whites. Journal of forensic sciences 2007;52:428-433. | Does not match PIRO |
|  | Boonpitaksathit T, Hunt N, Roberts GJ, Petrie A, Lucas VS. Dental age assessment of adolescents and emerging adults in United Kingdom Caucasians using censored data for stage H of third molar roots. European journal of orthodontics 2011;33:503-508. | Does not match PIRO |
|  | Boyacioglu Dogru H, Gulsahi A, Cehreli SB, Galic I, van der Stelt P, Cameriere R. Age of majority assessment in Dutch individuals based on Cameriere's third molar maturity index. Forensic science international 2018;282:231.e231-231.e236. | Does not match PIRO |
|  | Brotons A, Remy F, Foti B, Philip-Alliez C. Concordances and correlations between chronological, dental and bone ages: A retrospective study in French individuals. Forensic Sci Int.331:111150. | Does not match PIRO |
|  | Cameriere R, Brkic H, Ermenc B, Ferrante L, Ovsenik M, Cingolani M. The measurement of open apices of teeth to test chronological age of over 14-year olds in living subjects. Forensic science international 2008;174:217-221. | Does not match PIRO |
|  | Cameriere R, Ferrante L, Liversidge HM, Prieto JL, Brkic H. Accuracy of age estimation in children using radiograph of developing teeth. Forensic science international 2008;176:173-177. | Does not match PIRO |
|  | Cameriere R, Pacifici A, Viva S, Carbone D, Pacifici L, Polimeni A. Adult or not? Accuracy of Cameriere's cut-off value for third molar in assessing 18 years of age for legal purposes. Minerva stomatologica 2014;63:283-294. | Does not match PIRO |
|  | Cameriere R, Santoro V, Roca R, Lozito P, Introna F, Cingolani M, et al. Assessment of legal adult age of 18 by measurement of open apices of the third molars: Study on the Albanian sample. Forensic science international 2014;245:205.e201-205. | Does not match PIRO |
|  | Cameriere R, Velandia Palacio LA, Pinares J, Bestetti F, Paba R, Coccia E, et al. Assessment of second (I2M) and third (I3M) molar indices for establishing 14 and 16 legal ages and validation of the Cameriere's I3M cut-off for 18 years old in Chilean population. Forensic science international 2018;285:205.e201-205.e205. | Does not match PIRO |
|  | Cameriere R, Velandia Palacio LA, Marchetti M, Baralla F, Cingolani M, Ferrante L. Child brides: the age estimation problem in young girls. The Journal of forensic odonto-stomatology 2020;3:2-7. | Does not match PIRO |
|  | Cantekin K, Sekerci AE, Buyuk SK. Dental computed tomographic imaging as age estimation: morphological analysis of the third molar of a group of Turkish population. The American journal of forensic medicine and pathology 2013;34:357-362. | Does not match PIRO |
|  | Cantekin K, Ercan Sekerci A, Peduk K, Delikan E, Ozakar Ilday N, Demirbuga S, et al. Dental age assessment for different climatic regions. The American journal of forensic medicine and pathology 2014;35:197-200. | Does not match PIRO |
|  | Cardoso HFV, Caldas IM, Andrade M. Dental and skeletal maturation as simultaneous and separate predictors of chronological age in post-pubertal individuals: a preliminary study in assessing the probability of having attained 16 years of age in the living. Australian Journal of Forensic Sciences 2018;50:371-384. | Does not match PIRO |
|  | Carneiro JL, Caldas IM, Afonso A, Cardoso HFV. Is Demirjian's original method really useful for age estimation in a forensic context? Forensic science, medicine, and pathology 2015;11:216-221. | Does not match PIRO |
|  | Cavric J, Galic I, Vodanovic M, Brkic H, Gregov J, Viva S, et al. Third molar maturity index (I3M) for assessing age of majority in a black African population in Botswana. International journal of legal medicine 2016;130:1109-1120. | Does not match PIRO |
|  | Celik S, Zeren C, Celikel A, Yengil E, Altan A. Applicability of the Demirjian method for dental assessment of southern Turkish children. Journal of forensic and legal medicine 2014;25:1-5. | Does not match PIRO |
|  | Celikoglu M, Cantekin K, Ceylan I. Dental age assessment: the applicability of Demirjian method in eastern Turkish children. Journal of forensic sciences 2011;56:S220-222. | Does not match PIRO |
|  | Chaudhary MA, Liversidge HM. A radiographic study estimating age of mandibular third molars by periodontal ligament visibility. The Journal of forensic odonto-stomatology 2017;35:79-89. | Does not match PIRO |
|  | Chen JW, Guo J, Zhou J, Liu RK, Chen TT, Zou SJ. Assessment of dental maturity of western Chinese children using Demirjian's method. Forensic science international 2010;197:119.e111-114. | Does not match PIRO |
|  | Chhaparwal Y, Kumar M, Madi M, Chhaparwal S, Pentapati KC. Age estimation by modified demirjian’s method in a hospital-based population: A radiographic study. Pesquisa Brasileira em Odontopediatria e Clinica Integrada. 2021;21. | Does not match PIRO |
|  | Choudhury BK, Bhuyan SK, Pati A, Misra SR, Panigrahi R, Priyadarshini SR, et al. Estimation of CA using orocervical radiographic indices: A prospective observational study. Indian Journal of Forensic Medicine and Toxicology 2019;13:1846-1851. | Does not match PIRO |
|  | Chu G, Wang YH, Li MJ, Han MQ, Zhang ZY, Chen T, et al. Third molar maturity index (I3M) for assessing age of majority in northern Chinese population. International journal of legal medicine 2018;132:1759-1768. | Does not match PIRO |
|  | Chu G, Han MQ, Chen T, Zhou H, Guo YC. Construction of Age Estimation Model of 18-Year-Olds in the Northern Chinese Population Based on the Development of Mandibular Second and Third Molars. Fa yi xue za zhi 2019;35:289-294. | Language |
|  | Corradi F, Pinchi V, Barsanti I, Manca R, Garatti S. Optimal age classification of young individuals based on dental evidence in civil and criminal proceedings. International journal of legal medicine 2013;127:1157-1164. | Does not match PIRO |
|  | Corral C, García F, García J, León P, Herrera A, Martínez C, et al. Chronological versus dental age in subjects from 5 to 19 years: A comparative study with forensic implications. Colombia Medica 2010;41:215-223. | Does not match PIRO |
|  | Correia AdM, Barbosa DdS, Alcantara JAdS, Oliveira PMdC, Silva PGdB, Franco A, et al. Performance and comparison of the London Atlas technique and Cameriere's third molar maturity index (I3M) for allocating individuals below or above the threshold of 18 years. Forensic science international 2020;317:110512. | Does not match PIRO |
|  | Cruz-Landeira A, Linares-Argote J, Martinez-Rodriguez M, Rodriguez-Calvo MS, Otero XL, Concheiro L. Dental age estimation in Spanish and Venezuelan children. Comparison of Demirjian and Chaillet's scores. International journal of legal medicine 2010;124:105-112. | Does not match PIRO |
|  | Dardouri AAK, Cameriere R, De Luca S, Vanin S. Third molar maturity index by measurements of open apices in a Libyan sample of living subjects. Forensic science international 2016;267:230.e231-230.e236. | Does not match PIRO |
|  | Davidson CL, Nel C, Bernitz H, van Staden PJ, Uys A. Validation of Roberts' method using root canal width patterns as a mandibular maturity marker in determining the 18-year threshold. International journal of legal medicine 2021. | Does not match PIRO |
|  | De Angelis D, Gibelli D, Merelli V, Botto M, Ventura F, Cattaneo C. Application of age estimation methods based on teeth eruption: how easy is Olze method to use? International journal of legal medicine 2014;128:841-844. | Does not match PIRO |
|  | De Donno A, Angrisani C, Mele F, Introna F, Santoro V. Dental age estimation: Demirjian's versus the other methods in different populations. A literature review. Medicine, science, and the law 2021;61:125-129. | Does not match PIRO |
|  | De Luca S, Biagi R, Begnoni G, Farronato G, Cingolani M, Merelli V, et al. Accuracy of Cameriere's cut-off value for third molar in assessing 18 years of age. Forensic science international 2014;235:102.e101-106. | Does not match PIRO |
|  | De Luca S, Aguilar L, Rivera M, Palacio LAV, Riccomi G, Bestetti F, et al. Accuracy of cut-off value by measurement of third molar index: Study of a Colombian sample. Forensic science international 2016;261:160.e161-165. | Does not match PIRO |
|  | De Luca S, Pacifici A, Pacifici L, Polimeni A, Fischetto SG, Velandia Palacio LA, et al. Third molar development by measurements of open apices in an Italian sample of living subjects. Journal of forensic and legal medicine 2016;38:36-42. | Does not match PIRO |
|  | De Micco F, Martino F, Velandia Palacio LA, Cingolani M, Campobasso CP. Third molar maturity index and legal age in different ethnic populations: Accuracy of Cameriere's method. Medicine, science, and the law 2021;61:105-112. | Does not match PIRO |
|  | Deitos AR, Costa C, Michel-Crosato E, Galic I, Cameriere R, Biazevic MGH. Age estimation among Brazilians: Younger or older than 18? Journal of forensic and legal medicine 2015;33:111-115. | Does not match PIRO |
|  | Demirturk Kocasarac H, Sinanoglu A, Noujeim M, Helvacioglu Yigit D, Baydemir C. Radiologic assessment of third molar tooth and spheno-occipital synchondrosis for age estimation: a multiple regression analysis study. International journal of legal medicine 2016;130:799-808. | Does not match PIRO |
|  | Duangto P, Janhom A, Prasitwattanaseree S, Mahakkanukrauh P, Iamaroon A. New prediction models for dental age estimation in Thai children and adolescents. Forensic science international 2016;266:583.e581-583.e585. | Does not match PIRO |
|  | Esan TA, Yengopal V, Schepartz LA. The Demirjian versus the Willems method for dental age estimation in different populations: A meta-analysis of published studies. PloS one 2017;12:e0186682. | Does not match PIRO |
|  | Esan TA, Schepartz LA. Accuracy of the Demirjian and Willems methods of age estimation in a Black Southern African population. Legal medicine (Tokyo, Japan) 2018;31:82-89. | Does not match PIRO |
|  | Esan TA, Schepartz LA. The timing of permanent tooth development in a Black Southern African population using the Demirjian method. International journal of legal medicine 2019;133:257-268. | Does not match PIRO |
|  | Fan F, Dai Xh, Wang L, Li Y, Zhang K, Deng Zh. Establish Assessment Model of 18 Years of Age in Chinese Han Population by Mandibular Third Molar. Fa yi xue za zhi 2016;32:31-44. | Language |
|  | Fan Jl, Zhou Wl. Validity and reliability of the Demirjian distinguish software on estimating dental age. Zhongguo yi xue ke xue yuan xue bao. Acta Academiae Medicinae Sinicae 2005;27:363-366. | Language |
|  | Fei Y, Wang J. Study on development time of mandibular permanent teeth with Demirjian method in 928 children. Shanghai kou qiang yi xue = Shanghai journal of stomatology 2017;26:565-568. | Language |
|  | Franco A, de Oliveira MN, Campos Vidigal MT, Blumenberg C, Pinheiro AA, Paranhos LR. Assessment of dental age estimation methods applied to Brazilian children: a systematic review and meta-analysis. Dento maxillo facial radiology 2021;50:20200128. | Does not match PIRO |
|  | Franco RPAV, Franco A, Turkina A, Arakelyan M, Arzukanyan A, Velenko P, et al. Radiographic assessment of third molar development in a Russian population to determine the age of majority. Archives of oral biology 2021;125:105102. | Does not match PIRO |
|  | Franco RPAV, Franco A, Turkina A, Arakelyan M, Arzukanyan A, Velenko P, et al. Third molar classification using Gleiser and Hunt system modified by Khöler in Russian adolescents – Age threshold of 14 and 16. Forensic Imaging 2021;25. | Does not match PIRO |
|  | Franklin D, Karkhanis S, Flavel A, Collini F, DeLuca S, Cameriere R. Accuracy of a cut-off value based on the third molar index: Validation in an Australian population. Forensic science international 2016;266:575.e571-575.e576. | Does not match PIRO |
|  | Friedrich RE, Ulbricht C, Von Maydell LA, Scheuer HA. The impact of the topography of wisdom teeth on the chronology of root formation - Consequences for the forensic-odontologic age estimation of adolescents and young adults: Radiographic investigations of orthopantomograms. Archiv fur Kriminologie 2005;216:15-35. | Does not match PIRO |
|  | Galibourg A, Cussat-Blanc S, Dumoncel J, Telmon N, Monsarrat P, Maret D. Comparison of different machine learning approaches to predict dental age using Demirjian's staging approach. International journal of legal medicine 2021;135:665-675. | Does not match PIRO |
|  | Galic I, Lauc T, Brkic H, Vodanovic M, Galic E, Biazevic MGH, et al. Cameriere's third molar maturity index in assessing age of majority. Forensic science international 2015;252:191.e191-195. | Does not match PIRO |
|  | Gandhi N, Jain S, Kumar M, Rupakar P, Choyal K, Prajapati S. Reliability of third molar development for age estimation in Gujarati population: A comparative study. Journal of forensic dental sciences 2015;7:107-113. | Does not match PIRO |
|  | Garg N, Kathuria A, Srikant N, Nandita KP, Yellapurkar S, Jose NP, et al. Validity of Willems age estimation method in children’s & adolescents’ of Dakshina Kannada Region, India. Journal of the Canadian Society of Forensic Science 2021;54:27-33. | Does not match PIRO |
|  | Gelbrich B, Frerking C, Weiss S, Schwerdt S, Stellzig-Eisenhauer A, Tausche E, et al. Combining wrist age and third molars in forensic age estimation: how to calculate the joint age estimate and its error rate in age diagnostics. Annals of human biology 2015;42:389-396. | Does not match PIRO |
|  | Gilbert C, Fairgrieve SI, Keenan SC. A Test of the Demirjian method of dental ageing using a mixed population sample from Northern Ontario. Journal of the Canadian Society of Forensic Science 2014;47:1-19. | Does not match PIRO |
|  | Ginzelova K, Dostalova T, Eliasova H, Vinsu A, Bucek A, Buckova M. Using Dental Age to Estimate Chronological Age in Czech Children Aged 3-18 Years. Prague medical report 2015;116:139-154. | Does not match PIRO |
|  | Gomez Jimenez L, Velandia Palacio LA, De Luca S, Ramirez Vasquez Y, Corominas Capellan M, Cameriere R. Validation of the third molar maturity index (I3M): study of a Dominican Republic sample. The Journal of forensic odonto-stomatology 2019;3:27-33. | Does not match PIRO |
|  | Gonçalves LS, Machado ALR, Gaêta-Araujo H, Recalde TSF, Oliveira-Santos C, Silva RHAD. A comparison of Demirjian and Willems age estimation methods in a sample of Brazilian non-adult individuals. Forensic Imaging. 2021;25. | Does not match PIRO |
|  | Gulsahi A, Tirali RE, Cehreli SB, De Luca S, Ferrante L, Cameriere R. The reliability of Cameriere's method in Turkish children: a preliminary report. Forensic science international 2015;249:319.e311-315. | Does not match PIRO |
|  | Gulsahi A, De Luca S, Cehreli SB, Tirali RE, Cameriere R. Accuracy of the third molar index for assessing the legal majority of 18 years in Turkish population. Forensic science international 2016;266:584.e581-584.e586. | Does not match PIRO |
|  | Gungor OE, Kale B, Celikoglu M, Gungor AY, Sari Z. Validity of the Demirjian method for dental age estimation for Southern Turkish children. Nigerian journal of clinical practice 2015;18:616-619. | Does not match PIRO |
|  | Gunst K, Mesotten K, Carbonez A, Willems G. Third molar root development in relation to chronological age: a large sample sized retrospective study. Forensic science international 2003;136:52-57. | Does not match PIRO |
|  | Guo Yc, Yan Cx, Lin Xw, Zhou H, Pan F, Wei L, et al. Studies of the chronological course of third molars eruption in a northern Chinese population. Archives of oral biology 2014;59:906-911. | Does not match PIRO |
|  | Guo Y, Olze A, Ottow C, Schmidt S, Schulz R, Heindel W, et al. Dental age estimation in living individuals using 3.0 T MRI of lower third molars. International journal of legal medicine 2015;129:1265-1270. | Does not match PIRO |
|  | Guo YC, Chu G, Olze A, Schmidt S, Schulz R, Ottow C, et al. Application of age assessment based on the radiographic visibility of the root pulp of lower third molars in a northern Chinese population. International journal of legal medicine 2018;132:825-829. | Does not match PIRO |
|  | Guo YC, Wang YH, Olze A, Schmidt S, Schulz R, Pfeiffer H, et al. Dental age estimation based on the radiographic visibility of the periodontal ligament in the lower third molars: application of a new stage classification. International journal of legal medicine 2020;134:369-374. | Does not match PIRO |
|  | Gupta S, Mehendiratta M, Rehani S, Kumra M, Nagpal R, Gupta R. Age estimation in Indian children and adolescents in the NCR region of Haryana: A comparative study. Journal of forensic dental sciences 2015;7:253-258. | Does not match PIRO |
|  | Haglund M, Mornstad H. A systematic review and meta-analysis of the fully formed wisdom tooth as a radiological marker of adulthood. International journal of legal medicine 2019;133:231-239. | A systematic review with high risk of bias |
|  | Hegde RJ, Khare SS, Saraf TA, Trivedi S, Naidu S. Evaluation of the accuracy of Demirjian method for estimation of dental age among 6-12 years of children in Navi Mumbai: A radiographic study. Journal of the Indian Society of Pedodontics and Preventive Dentistry 2015;33:319-323. | Does not match PIRO |
|  | Hegde S, Patodia A, Dixit U. Staging of third molar development in relation to chronological age of 5-16-year-old Indian children. Forensic science international 2016;269:63-69. | Does not match PIRO |
|  | Hegde S, Patodia A, Dixit U. A comparison of the validity of the Demirjian, Willems, Nolla and Haavikko methods in determination of chronological age of 5-15-year-old Indian children. Journal of forensic and legal medicine 2017;50:49-57. | Does not match PIRO |
|  | Hegde S, Patodia A, Shah K, Dixit U. The applicability of the Demirjian, Willems and Chaillet standards to age estimation of 5-15-year-old Indian children. The Journal of forensic odonto-stomatology 2019;37:40-50. | Does not match PIRO |
|  | Hofmann E, Robold M, Proff P, Kirschneck C. Age assessment based on third molar mineralisation : An epidemiological-radiological study on a Central-European population. Altersbestimmung anhand der Weisheitszahnmineralisation : Eine epidemiologisch-radiologische Studie an einem mitteleuropaischen Probandengut. 2017;78:97-111. | Does not match PIRO |
|  | Ifesanya JU, Adeyemi AT. Accuracy of age estimation using Demirjian method among Nigerian children. African journal of medicine and medical sciences 2012;41:297-300. | Does not match PIRO |
|  | Jafari A, Mohebbi S, Khami M, Shahabi MS, Naseh M, Elhami F, et al. Radiographic evaluation of third molar development in 5- to 25-year olds in tehran, iran. Journal of dentistry (Tehran, Iran) 2012;9:107-115. | Does not match PIRO |
|  | Javadinejad S, Sekhavati H, Ghafari R. A Comparison of the Accuracy of Four Age Estimation Methods Based on Panoramic Radiography of Developing Teeth. Journal of dental research, dental clinics, dental prospects 2015;9:72-78. | Does not match PIRO |
|  | Jayaraman J, Roberts GJ, King NM, Wong HM. Dental age assessment of southern Chinese using the United Kingdom Caucasian reference dataset. Forensic science international 2012;216:68-72. | Does not match PIRO |
|  | Jayaraman J, Wong HM, King NM, Roberts GJ. The French-Canadian data set of Demirjian for dental age estimation: a systematic review and meta-analysis. Journal of forensic and legal medicine 2013;20:373-381. | Does not match PIRO |
|  | Jayaraman J, Roberts G. Demirjian's method is unsuitable for dental age estimation. Forensic science, medicine, and pathology 2016;12:532-533. | Does not match PIRO |
|  | Jayaraman J, Roberts GJ. Comparison of dental maturation in Hong Kong Chinese and United Kingdom Caucasian populations. Forensic science international 2018;292:61-70. | Does not match PIRO |
|  | Jin H, Cheng M, Liu H, Chen Y, Ou G, Zhao H. Dental age assessment of the third molar using demirjian's method in 344 teenagers of guangdong han population and the application of forensic science. Chinese Journal of Forensic Medicine 2014;29:194-197. | Does not match PIRO |
|  | Kanchan T, Chugh V, Chugh A, Meshram V, Shedge R, Patnana AK, et al. Age estimation using third molar maturation based on Demirjian's criteria. Leg Med (Tokyo).53:101959. | Does not match PIRO |
|  | Kar May L, Mei Shian AY, Durward C, Jayaraman J. A method of estimating age of undocumented children and young adults of different socioeconomic status in Cambodia. Heliyon 2020;6:e03476. | Does not match PIRO |
|  | Karadayi B, Kaya A, Kolusayin MO, Karadayi S, Afsin H, Ozaslan A. Radiological age estimation: based on third molar mineralization and eruption in Turkish children and young adults. International journal of legal medicine 2012;126:933-942. | Does not match PIRO |
|  | Karadayi B, Afsin H, Ozaslan A, Karadayi S. Development of dental charts according to tooth development and eruption for Turkish children and young adults. Imaging science in dentistry 2014;44:103-113. | Does not match PIRO |
|  | Karatas OH, Ozturk F, Dedeoglu N, Colak C, Altun O. Radiographic evaluation of third-molar development in relation to the chronological age of Turkish children in the southwest Eastern Anatolia region. Forensic science international 2013;232:238.e231-235. | Does not match PIRO |
|  | Karimi A, Qudeimat MA, Lucas VS, Roberts G. Dental age estimation: Development and validation of a reference data set for Kuwaiti children, adolescents, and young adults. Arch Oral Biol.127:105130. | Does not match PIRO |
|  | Kedarisetty SG, Rao GV, Rayapudi N, Korlepara R. Evaluation of skeletal and dental age using third molar calcification, condylar height and length of the mandibular body. Journal of forensic dental sciences 2015;7:121-125. | Does not match PIRO |
|  | Kelmendi J, Cameriere R, Kocani F, Galic I, Mehmeti B, Vodanovic M. The third molar maturity index in indicating the legal adult age in Kosovar population. International journal of legal medicine 2018;132:1151-1159. | Does not match PIRO |
|  | Kermani M, Tabatabaei Yazdi F, Abed Haghighi M. Evaluation of the accuracy of Demirjian's method for estimating chronological age from dental age in Shiraz, Iran: Using geometric morphometrics method. Clinical and experimental dental research 2019;5:191-198. | Does not match PIRO |
|  | Khare P, Li J, Velandia Palacio LA, Galic I, Ferrante L, Cameriere R. Validation of the third molar maturity index cut-off value of <0.08 for indicating legal age of 18 years in Eastern Chinese region. Legal medicine (Tokyo, Japan) 2020;42:101645. | Does not match PIRO |
|  | Khdairi N, Halilah T, Khandakji MN, Jost-Brinkmann PG, Bartzela T. The adaptation of Demirjian's dental age estimation method on North German children. Forensic science international 2019;303:109927. | Does not match PIRO |
|  | Khoja A, Fida M, Shaikh A. Validity of different dental age estimation methods in Pakistani orthodontic patients. Australian Journal of Forensic Sciences 2015;47:283-292. | Does not match PIRO |
|  | Khorate MM, Dinkar AD, Ahmed J. Accuracy of age estimation methods from orthopantomograph in forensic odontology: a comparative study. Forensic science international 2014;234:184.e181-188. | Does not match PIRO |
|  | Kiran CS, Reddy RS, Ramesh T, Madhavi NS, Ramya K. Radiographic evaluation of dental age using Demirjian's eight-teeth method and its comparison with Indian formulas in South Indian population. Journal of forensic dental sciences 2015;7:44-48. | Does not match PIRO |
|  | Konigsberg LW, Frankenberg SR, Liversidge HM. Status of Mandibular Third Molar Development as Evidence in Legal Age Threshold Cases. Journal of forensic sciences 2019;64:680-697. | Does not match PIRO |
|  | Kullman L. Accuracy of two dental and one skeletal age estimation method in Swedish adolescents. Forensic science international 1995;75:225-236. | Does not match PIRO |
|  | Kumagai A, Willems G, Franco A, Thevissen P. Age estimation combining radiographic information of two dental and four skeletal predictors in children and subadults. International journal of legal medicine 2018;132:1769-1777. | Does not match PIRO |
|  | Kumagai A, Takahashi N, Palacio LAV, Giampieri A, Ferrante L, Cameriere R. Accuracy of the third molar index cut-off value for estimating 18years of age: Validation in a Japanese samples. Legal medicine (Tokyo, Japan) 2019;38:5-9. | Does not match PIRO |
|  | Kumar GK, Kumar DRS, Kulkarni G, Balla SB, Shyam NDVN, Naishadham Y. Olze et al. stages of radiographic visibility of root pulp and cameriere's third molar maturity index to estimate legal adult age in Hyderabad population. Journal of forensic dental sciences 2019;11:84-89. | Does not match PIRO |
|  | Kumar VJ, Gopal KS. Reliability of age estimation using Demirjian's 8 teeth method and India specific formula. Journal of forensic dental sciences 2011;3:19-22. | Does not match PIRO |
|  | Kumaresan R, Cugati N, Chandrasekaran B, Karthikeyan P. Reliability and validity of five radiographic dental-age estimation methods in a population of Malaysian children. Journal of investigative and clinical dentistry 2016;7:102-109. | Does not match PIRO |
|  | Kutesa AM, Rwenyonyi CM, Mwesigwa CL, Muhammad M, Nabaggala GS, Kalyango J. Dental age estimation using radiographic assessment of third molar eruption among 10-20-year-old Ugandan population. Journal of forensic dental sciences 2019;11:16-21. | Does not match PIRO |
|  | Lan LM, Yang ZD, Sun SL, Wen D, Kureshi A, Zeye MMJ, et al. Application of Demirjian's and Cameriere's Method in Dental Age Estimation of 8-16-Year-Old Adolescents from Hunan Han Nationality. Fa yi xue za zhi 2019;35:406-410. | Language |
|  | Lee SS, Kim D, Lee S, Lee UY, Seo JS, Ahn YW, et al. Validity of Demirjian's and modified Demirjian's methods in age estimation for Korean juveniles and adolescents. Forensic science international 2011;211:41-46. | Does not match PIRO |
|  | Lee Yin ET, Sim KP, Putera Mohd Yusof MY. Adaptation of Demirjian’s method for age estimation via third molar development among adolescents and young adults of Malay ethnicity: A preliminary assessment. Malaysian Journal of Medicine and Health Sciences 2020;16:14-18. | Does not match PIRO |
|  | Litsas G, Athanasiou AE, Papadopoulos MA, Ioannidou-Marathiotou I, Karagiannis V. Dental calcification stages as determinants of the peak growth period. Phasen in der dentalen Kalzifikation als Determinanten der Hauptwachstumsperiode. 2016;77:341-349. | Does not match PIRO |
|  | Liversidge HM, Marsden PH. Estimating age and the likelihood of having attained 18 years of age using mandibular third molars. British dental journal 2010;209:E13. | Does not match PIRO |
|  | Liversidge HM. Interpreting group differences using Demirjian's dental maturity method. Forensic science international 2010;201:95-101. | Does not match PIRO |
|  | Liversidge HM, Smith H. Nolla's longitudinal dental study revisited. American Journal of Physical Anthropology 2014;153:171. | Does not match PIRO |
|  | Liversidge HM, Konigsberg LW. Recent analytical developments yield new insights into the timing of tooth formation and standards for age estimation. American Journal of Physical Anthropology 2016;159:210-211. | Does not match PIRO |
|  | Liversidge HM, Peariasamy K, Folayan MO, Adeniyi AO, Ngom PI, Mikami Y, et al. A radiographic study of the mandibular third molar root development in different ethnic groups. The Journal of forensic odonto-stomatology 2017;35:97-108. | Does not match PIRO |
|  | Lu M, Lai X, Tan Q, Ding Z, Deng M, Liang C, et al. Dental calcification of 15-22 years old male in Dongguan city and its forensic significance. Chinese Journal of Forensic Medicine 2013;28:310-313. | Does not match PIRO |
|  | Maled V, Manjunatha B, Patil K, Balaraj BM. The chronology of third molar root mineralization in south Indian population. Medicine, science, and the law 2014;54:28-34. | Does not match PIRO |
|  | Mardiati E, Komara I, Halim H, Kurnia D, Maskoen AM. Sensitivity and specificity of mandibular third molar calcification at chronological age and hand wrist maturation stage to discriminate between female and male at pubertal growth period. Open Dentistry Journal. 2021;15(1):551-7. | Does not match PIRO |
|  | Marquez-Ruiz AB, Trevino-Tijerina MC, Gonzalez-Herrera L, Sanchez B, Gonzalez-Ramirez AR, Valenzuela A. Three-dimensional analysis of third molar development to estimate age of majority. Science & justice : journal of the Forensic Science Society 2017;57:376-383. | Does not match PIRO |
|  | Marrero-Ramos MD, Lopez-Urquia L, Suarez-Soto A, Sanchez-Villegas A, Vicente-Barrero M. Estimation of the age of majority through radiographic evaluation of the third molar maturation degree. Medicina oral, patologia oral y cirugia bucal 2020;25:e359-e363. | Does not match PIRO |
|  | Mauricio-Vilchez C, Mauricio F, Vilchez L, Cadenillas A, Medina J, Mayta-Tovalino F. Radiographic Correlation of Skeletal Maturation Using the Stages of Dental Calcification in a Peruvian Population. Scientifica 2020;2020:4052619. | Does not match PIRO |
|  | McLelland E, Keenan S, Fairgrieve SI. A cross-sectional study of three dental age estimation techniques applied to permanent mandibular teeth in a Northern Ontario population and their reproducibility in forensic investigations. Journal of the Canadian Society of Forensic Science 2019;52:18-19. | Does not match PIRO |
|  | Medina AC, Blanco L. Accuracy of dental age estimation in Venezuelan children: comparison of Demirjian and Willems methods. Acta odontologica latinoamericana : AOL 2014;27:34-41. | Does not match PIRO |
|  | Meghana RV, Mallempalli P, Kondakamalli S, Boringi M, Vaddeswarapu RM, Kairamkonda CR, et al. A test to study the influence of impaction on mandibular third molar development and forensic age estimation in a sample of south Indian children and young adults. Leg Med (Tokyo).54:101998. | Does not match PIRO |
|  | Melo M, Ata-Ali J. Accuracy of the estimation of dental age in comparison with chronological age in a Spanish sample of 2641 living subjects using the Demirjian and Nolla methods. Forensic science international 2017;270:276.e271-276.e277. | Does not match PIRO |
|  | Meshram AH, Dode CR, Lanjewar DN. Estimation of age of Indian adolescents by radiographic study of mandibular third molar. Indian Journal of Forensic Medicine and Toxicology 2013;7:246-250. | Does not match PIRO |
|  | Mesotten K, Gunst K, Carbonez A, Willems G. Dental age estimation and third molars: a preliminary study. Forensic science international 2002;129:110-115. | Does not match PIRO |
|  | Metsaniitty M, Waltimo-Siren J, Ranta H, Fieuws S, Thevissen P. Dental age estimation in Somali children using the Willems et al. model. International journal of legal medicine 2018;132:1779-1786. | Does not match PIRO |
|  | Metsaniitty M, Waltimo-Siren J, Ranta H, Fieuws S, Thevissen P. Dental age estimation in Somali children and sub-adults combining permanent teeth and third molar development. International journal of legal medicine 2019;133:1207-1215. | Does not match PIRO |
|  | Mishra SS, Parakh A. Accuracy of Chaillet and Demirjian's 8-teeth method in dental age estimation of Central Indian population using India specific regression formulae. Forensic Science International: Reports 2020;2. | Does not match PIRO |
|  | Mitchell JC, Roberts GJ, Donaldson ANA, Lucas VS. Dental age assessment (DAA): reference data for British caucasians at the 16-year threshold. Forensic science international 2009;189:19-23. | Does not match PIRO |
|  | Mohammed RB, Sanghvi P, Perumalla KK, Srinivasaraju D, Srinivas J, Kalyan US, et al. Accuracy of four dental age estimation methods in southern Indian children. Journal of clinical and diagnostic research : JCDR 2015;9:HC01-08. | Does not match PIRO |
|  | Mohammed RB, Srinivas B, Sanghvi P, Satyanarayana G, Gopalakrishnan M, Pavani BV. Accuracy of Demirjian's 8 teeth method for age prediction in South Indian children: A comparative study. Contemporary clinical dentistry 2015;6:5-11. | Does not match PIRO |
|  | Mohan R, Jain RK, Balakrishnan N. Assessment of growth status by correlating the maturation stages of middle phalanx of the third finger and calcification stages of mandibular third molar in an institutional set up. International Journal of Research in Pharmaceutical Sciences 2020;11:1965-1969. | Does not match PIRO |
|  | Mohanty I, Panda S, Dalai RP, Mohanty N. Predictive accuracy of Demirjian's, Modified Demirjian's and India specific dental age estimation methods in Odisha (Eastern Indian) population. The Journal of forensic odonto-stomatology 2019;37:32-39. | Does not match PIRO |
|  | Mohd Yusof MYP, Cauwels R, Deschepper E, Martens L. Application of third molar development and eruption models in estimating dental age in Malay sub-adults. Journal of forensic and legal medicine 2015;34:40-44. | Does not match PIRO |
|  | Mohd Yusof MYP, Cauwels R, Martens L. Stages in third molar development and eruption to estimate the 18-year threshold Malay juvenile. Archives of oral biology 2015;60:1571-1576. | Does not match PIRO |
|  | Mohd Yusof MYP, Wan Mokhtar I, Rajasekharan S, Overholser R, Martens L. Performance of Willem's dental age estimation method in children: A systematic review and meta-analysis. Forensic science international 2017;280:245.e241-245.e210. | Does not match PIRO |
|  | Monirifard M, Yaraghi N, Vali A, Vali A, Vali A. Radiographic assessment of third molars development and it's relation to dental and chronological age in an Iranian population. Dental research journal 2015;12:64-70. | Does not match PIRO |
|  | Moode PK, Kumar LA, Mittapelly R. Accuracy of Demerjian age estimation method in south India KADAPA [A.P] population-a cross sectional study. Medico-Legal Update 2020;20:175-179. | Does not match PIRO |
|  | Moukarzel M, Angelakopoulos N, De Luca S, Velandia Palacio LA, Aquilanti L, Coccia E, et al. Validity assessment of the third molar maturity index (I3M) in a Lebanese sample of adolescents and young adults. Australian Journal of Forensic Sciences 2020. | Does not match PIRO |
|  | Ndiaye ML, Soumboundou S, Douch H, Lecor PA, Ly-Ba A, Toure B. Demirjian's stages and Camérière's third molar maturity index to estimate legal adult age in Senegalese population. Revue de Medecine Legale 2020;11:150-157. | Language |
|  | Nik-Hussein NN, Kee KM, Gan P. Validity of Demirjian and Willems methods for dental age estimation for Malaysian children aged 5-15 years old. Forensic science international 2011;204:208.e201-206. | Does not match PIRO |
|  | Nobrega JBMd, Protasio APL, Ribeiro ILA, Valenca AMG, Santiago BM, Cameriere R. Validation of the Third Molar Maturation Index to estimate the age of criminal responsibility in Northeastern Brazil. Forensic science international 2019;304:109917. | Does not match PIRO |
|  | Nour El Deen REH, Alduaiji HM, Alajlan GM, Aljabr AA. Development of the Permanent Dentition and Validity of Demirjian and Goldstein Method for Dental Age Estimation in Sample of Saudi Arabian Children (Qassim Region). International journal of health sciences 2016;10:21-28. | Does not match PIRO |
|  | Nystrom ME, Ranta HM, Peltola JS, Kataja JM. Timing of developmental stages in permanent mandibular teeth of Finns from birth to age 25. Acta odontologica Scandinavica 2007;65:36-43. | Does not match PIRO |
|  | Olze A, Schmeling A, Rieger K, Kalb G, Geserick G. Studies on the chronology of third molar mineralization in a German population. Rechtsmedizin 2003;13:5-10. | Does not match PIRO |
|  | Olze A, Bilang D, Schmidt S, Wernecke KD, Geserick G, Schmeling A. Validation of common classification systems for assessing the mineralization of third molars. International journal of legal medicine 2005;119:22-26. | Does not match PIRO |
|  | Olze A, Hertel J, Schulz R, Wierer T, Schmeling A. Radiographic evaluation of Gustafson's criteria for the purpose of forensic age diagnostics. International journal of legal medicine 2012;126:615-621. | Does not match PIRO |
|  | Ottow C, Krämer JA, Olze A, Schmidt S, Schulz R, Wittschieber D, et al. Magnetic resonance tomography studies on age estimation of unaccompanied minor refugees. Rechtsmedizin 2015;25:12-20. | Does not match PIRO |
|  | Ozveren N, Serindere G. Comparison of the applicability of Demirjian and Willems methods for dental age estimation in children from the Thrace region, Turkey. Forensic science international 018;285:38-43. | Does not match PIRO |
|  | Palanisamy V, Rao A, Shenoy R, Baranya SS. Correlation of dental age, skeletal age, and chronological age among children aged 9-14 years: A retrospective study. Journal of the Indian Society of Pedodontics and Preventive Dentistry 2016;34:310-314. | Does not match PIRO |
|  | Palmela Pereira C, Rodrigues A, Santos A, Salvado F, Santos R, Cameriere R. Cut-off for the legal ages in the Portuguese Population by Third Maturity Index: Measures of Accuracy. Archives of oral biology 2021;125:105089. | Does not match PIRO |
|  | Pan J, Shen C, Yang Z, Fan L, Wang M, Shen S, et al. A modified dental age assessment method for 5- to 16-year-old eastern Chinese children. Clinical oral investigations 2021. | Does not match PIRO |
|  | Panainte I, Pop SI, Martha K. Correlation Among Chronological Age, Dental Age and Cervical Vertebrae Maturity in Romanian Subjects. Revista medico-chirurgicala a Societatii de Medici si Naturalisti din Iasi 2016; 120:700-710. | Does not match PIRO |
|  | Pandey H, Tripathi V, Pathak H, Choudhary SK, Parchake MB. Age estimation and comparison by dental and skeletal maturity in the age range of 9-18 years in the Mumbai region. Journal of forensic dental sciences 2019;11:142-146. | Does not match PIRO |
|  | Patel PS, Chaudhary AR, Dudhia BB, Bhatia PV, Soni NC, Jani YV. Accuracy of two dental and one skeletal age estimation methods in 6-16-year-old Gujarati children. Journal of forensic dental sciences 2015;7:18-27. | Does not match PIRO |
|  | Patnana AK, Vabbalareddy RS, V Vanga NR. Evaluating the reliability of three different dental age estimation methods in visakhapatnam children. International journal of clinical pediatric dentistry 2014;7:186-191. | Does not match PIRO |
|  | Patnana AK, Vanga NRV, Chandrabhatla SK, Vabbalareddy R. Dental age estimation using percentile curves and regression analysis methods - A test of accuracy and reliability. Journal of Clinical and Diagnostic Research 2018;12:ZC01-ZC04. | Does not match PIRO |
|  | Pavlovic S, Palmela Pereira C, Vargas de Sousa Santos RF. Age estimation in Portuguese population: The application of the London atlas of tooth development and eruption. Forensic science international 2017;272:97-103. | Does not match PIRO |
|  | Peiris TS, Roberts GJ, Prabhu N. Dental Age Assessment: a comparison of 4- to 24-year-olds in the United Kingdom and an Australian population. International journal of paediatric dentistry 2009;19:367-376. | Does not match PIRO |
|  | Periyakaruppan S, Meundi MA, David CM. Accuracy of age estimation in 6-21-year-old South Indian population - A comparative analysis of clinical and radiographic methods. The Journal of forensic odonto-stomatology 2018;36:10-19. | Does not match PIRO |
|  | Phillips VM, van Wyk Kotze TJ. Testing standard methods of dental age estimation by Moorrees, Fanning and Hunt and Demirjian, Goldstein and Tanner on three South African children samples. The Journal of forensic odonto-stomatology 2009;27:20-28. | Does not match PIRO |
|  | Pinchi V, De Luca F, Focardi M, Pradella F, Vitale G, Ricciardi F, et al. Combining dental and skeletal evidence in age classification: Pilot study in a sample of Italian sub-adults. Legal medicine (Tokyo, Japan) 2016;20:75-79. | Does not match PIRO |
|  | Pinchi V, Pradella F, Vitale G, Rugo D, Nieri M, Norelli GA. Comparison of the diagnostic accuracy, sensitivity and specificity of four odontological methods for age evaluation in Italian children at the age threshold of 14 years using ROC curves. Medicine, science, and the law 2016;56:13-18. | Does not match PIRO |
|  | Pourtaji B, Nasiri A. Journal of Zanjan University of Medical Sciences and Health Services 2017;25:104-114. | Does not match PIRO |
|  | Prabhakar AR, Panda AK, Raju OS. Applicability of Demirjian's method of age assessment in children of Davangere. Journal of the Indian Society of Pedodontics and Preventive Dentistry 2002;20:54-62. | Does not match PIRO |
|  | Prasad H, Kala N. Accuracy of two dental age estimation methods in the Indian population - A meta-analysis of published studies. The Journal of forensic odonto-stomatology 2019;3:2-11. | Does not match PIRO |
|  | Priyanka JN, Chaitanya N, Srivani GS, Mounika Y, Reddy GR, Priya B, et al. Correlating the Age Estimated by Nolla's Method and Modified Demirjian Method with Cervical Vertebral Maturation Index: A Cross-Sectional Study. Journal of the International Clinical Dental Research Organization.12(2):132-9. | Does not match PIRO |
|  | Putri AS, Soedarsono N, Nehemia B, Atmadja DS, Ubelaker DH. Age estimation of individuals aged 5–23 years based on dental development of the Indonesian population. Forensic Sciences Research. 2021. | Does not match PIRO |
|  | Pyata JR, ukuri BA, Gangavarapu U, Anjum B, Chinnala B, Bojji M, et al. Accuracy of four dental age estimation methods in determining the legal age threshold of 18 years among South Indian adolescents and young. J Forensic Odontostomatol.3(39):2-15. | Does not match PIRO |
|  | Rai V, Saha S, Yadav G, Tripathi AM, Grover K. Dental and skeletal maturity- a biological indicator of chronologic age. Journal of clinical and diagnostic research : JCDR 2014;8:ZC60-64. | Does not match PIRO |
|  | Ranasinghe S, Perera J, Taylor JA, Tennakoon A, Pallewatte A, Jayasinghe R. Dental age estimation using radiographs: Towards the best method for Sri Lankan children. Forensic science international 2019;298:64-70. | Does not match PIRO |
|  | Rath H, Rath R, Mahapatra S, Debta T. Assessment of Demirjian's 8-teeth technique of age estimation and Indian-specific formulas in an East Indian population: A cross-sectional study. Journal of forensic dental sciences 2017;9:45. | Does not match PIRO |
|  | Ribier L, Saint-Martin P, Seignier M, Pare A, Brunereau L, Rerolle C. Cameriere's third molar maturity index in assessing age of majority: a study of a French sample. International journal of legal medicine 2020;134:783-792. | Does not match PIRO |
|  | Roberts GJ, Lucas VS, Andiappan M, McDonald F. Dental Age Estimation: Pattern Recognition of Root Canal Widths of Mandibular Molars. A Novel Mandibular Maturity Marker at the 18-Year Threshold. Journal of forensic sciences 2017;62:351-354. | Does not match PIRO |
|  | Roberts GJ, McDonald F, Andiappan M, Lucas VS. Dental Age Estimation (DAE): Data management for tooth development stages including the third molar. Appropriate censoring of Stage H, the final stage of tooth development. Journal of forensic and legal medicine 2015;36:177-184. | Does not match PIRO |
|  | Rodríguez A, Verdugo V, Loarte G, Villavicencio E, Torracchi E. Estimation of the chronological age based on the mineralization of the third lower molar in the andean population. Revista Estomatologica Herediana 2020;30:272-277. | Does not match PIRO |
|  | Rolseth V, Mosdol A, Dahlberg PS, Ding Y, Bleka O, Skjerven-Martinsen M, et al. Age assessment by Demirjian's development stages of the third molar: a systematic review. European radiology 2019;29:2311-2321. | Does not match PIRO |
|  | Rolseth V, Mosdøl A, Dalberg PS, Ding KY, Bleka Ø, Skjerven‐Martinsen M, et al. Demirjian’s Development Stages on Wisdom Teeth for Estimation of Chronological Age: A Systematic Review; 2017. | Does not match PIRO |
|  | Rozkovcova E, Dostalova T, Markova M, Broukal Z. The third molar as an age marker in adolescents: new approach to age evaluation. Journal of forensic sciences 2012;57:1323-1328. | Does not match PIRO |
|  | Rozylo-Kalinowska I, Kalinowski P, Kozek M, Galic I, Cameriere R. Validity of the third molar maturity index I3M for indicating the adult age in the Polish population. Forensic science international 2018;290:352.e351-352.e356. | Does not match PIRO |
|  | Saade A, Baron P, Noujeim Z, Azar D. Dental and Skeletal Age Estimations in Lebanese Children: A Retrospective Cross-sectional Study. Journal of International Society of Preventive & Community Dentistry 2017;7:90-97. | Does not match PIRO |
|  | Santiago BM, Almeida L, Cavalcanti YW, Magno MB, Maia LC. Accuracy of the third molar maturity index in assessing the legal age of 18 years: a systematic review and meta-analysis. International journal of legal medicine 2018;132:1167-1184. | Does not match PIRO |
|  | Sarkar S, Kailasam S, Mahesh Kumar P. Accuracy of estimation of dental age in comparison with chronological age in Indian population--a comparative analysis of two formulas. Journal of forensic and legal medicine 2013;20:230-233. | Does not match PIRO |
|  | Sasso A, Spalj S, Mady Maricic B, Sasso A, Cabov T, Legovic M. Secular trend in the development of permanent teeth in a population of Istria and the littoral region of Croatia. Journal of forensic sciences 2013;58:673-677. | Does not match PIRO |
|  | Sasso A, Legovic M, Mady Maricic B, Pavlic A, Spalj S. Secular trend of earlier onset and decelerated development of third molars: evidence from Croatia. Forensic science international 2015;249:202-206. | Does not match PIRO |
|  | Scendoni R, Zolotenkova GV, Vanin S, Pigolkin YI, Cameriere R. Forensic Validity of the Third Molar Maturity Index (I 3M) for Age Estimation in a Russian Population. BioMed research international 2020;2020:6670590. | Does not match PIRO |
|  | Scheurer E, Sunitsch S, Stollberger R, Petrovic A. Dental age estimation: Evaluation of MR sequences for the imaging of tooth development. Rechtsmedizin 2011;21:364. | Does not match PIRO |
|  | Sehrawat JS, Singh M. Willems method of dental age estimation in children: A systematic review and meta-analysis. Journal of forensic and legal medicine 2017;52:122-129. | Does not match PIRO |
|  | Selmanagic A, Ajanovic M, Kamber-Cesir A, Redzepagic-Vrazalica L, Jeleskovic A, Nakas E. Radiological Evaluation of Dental Age Assessment Based on the Development of Third Molars in Population of Bosnia and Herzegovina. Acta stomatologica Croatica 2020;54:161-167. | Does not match PIRO |
|  | Shah R, Angadi PV. Radiographic assessment of periodontal ligament visibility in mandibular third molars as a tool for defining the 18-year threshold among Indians. Australian Journal of Forensic Sciences 2020. | Does not match PIRO |
|  | Sharma P, Wadhwan V, Ravi Prakash SM, Aggarwal P, Sharma N. Assessment of age of majority by measurement of open apices of the third molars using Cameriere's third molar maturity index. Journal of forensic dental sciences 2017;9:96-101. | Does not match PIRO |
|  | Sharma P, Wadhwan V, Sharma N. Reliability of determining the age of majority: a comparison between measurement of open apices of third molars and Demirjian stages. The Journal of forensic odonto-stomatology 2018;36:2-9. | Does not match PIRO |
|  | Sharma R, Srivastava A. Radiographic evaluation of dental age of adults using Kvaal's method. Journal of forensic dental sciences 2010;2:22-26. | Does not match PIRO |
|  | Shen C, Pan J, Yang Z, Mou H, Tao J, Ji F. Applicability of 2 Dental Age Estimation Methods to Taiwanese Population. The American journal of forensic medicine and pathology 2020;41:269-275. | Does not match PIRO |
|  | Sheta AA, Enany NM, Salama NH, Ahmed RA, Haiba MIM. Applicability of the modified Demirjian's method for age estimation in a sample of Egyptian children using dental radiography Modified Demirjian's method for age estimation. Annals of Clinical and Analytical Medicine.12:456-60. | Does not match PIRO |
|  | Shi GF, Lie RJ, Tao J, Fan LH, Zhu GY. Application of Demirjian's method for chronological age estimation in teenagers of Shanghai Han population. Fa yi xue za zhi 2009;25:168-171. | Language |
|  | Shilpa PH, Sunil RS, Sapna K, Kumar NC. Estimation and comparison of dental, skeletal and chronologic age in Bangalore south school going children. Journal of the Indian Society of Pedodontics and Preventive Dentistry 2013;31:63-68. | Does not match PIRO |
|  | Shivakumar B, Arunakshi A, Niveditha N, Shivaprasad S, Manjuprasad M. Validation of demirjian's 8-teeth method of age estimation in the population of Bengaluru. Journal of Oral and Maxillofacial Pathology. 2021;25(3):499-502. | Does not match PIRO |
|  | Soares CBRB, Figueiroa JN, Dantas RMX, Kurita LM, Pontual AdA, Ramos-Perez FMdM, et al. Evaluation of third molar development in the estimation of chronological age. Forensic science international 2015;254:13-17. | Does not match PIRO |
|  | Sobieska E, Fester A, Nieborak M, Zadurska M. Assessment of the Dental Age of Children in the Polish Population with Comparison of the Demirjian and the Willems Methods. Medical science monitor : international medical journal of experimental and clinical research 2018;24:8315-8321. | Does not match PIRO |
|  | Sousa AMdS, Jacometti V, AlQahtani S, Silva RHAd. Age estimation of Brazilian individuals using the London Atlas. Archives of oral biology 2020;113:104705. | Does not match PIRO |
|  | Spinas E, De Luca S, Lampis L, Velandia Palacio LA, Cameriere R. Is the third molar maturity index (I3M) useful for a genetic isolate population? Study of a Sardinian sample of children and young adults. International journal of legal medicine 2018;132:1787-1794. | Does not match PIRO |
|  | Srkoc T, Mestrovic S, Anic-Milosevic S, Slaj M. Association between Dental and Skeletal Maturation Stages in Croatian Subjects. Acta clinica Croatica 2015;54:445-452. | Does not match PIRO |
|  | Subedi N, Parajuli U, Paudel IS, Mallik M. Demirjian's Eight Teeth Method for Dental age Estimation in Nepalese Population. Journal of Nepal Health Research Council 2021;18:686-691. | Does not match PIRO |
|  | Sudha R, Balla SB. Usefulness of Demirjian's stages in determining the age of majority: A study on South Indian population. Journal of Indian Academy of Forensic Medicine 2017;39:123-130. | Does not match PIRO |
|  | Sujir N, Chauhan NP, Pai KM, Ahmed J, Denny C, Shenoy N. Radiographic evaluation of third molar development in relation to chronological age among South Indian population. Indian Journal of Forensic Medicine and Toxicology 2020;14:252-256. | Does not match PIRO |
|  | Sukhia RH, Fida M. Correlation among chronologic age, skeletal maturity, and dental age. World journal of orthodontics 2010;11:e78-84. | Does not match PIRO |
|  | Sybil D, Rai A, Kaur M, Mohanan M, Khatter H. Comparison of demirjian, nolla and cameriere's technique of age estimation using third molar teeth – a pilot study. Journal of Punjab Academy of Forensic Medicine and Toxicology. 2020;20(2):56-60. | Does not match PIRO |
|  | Tafrount C, Galic I, Franchi A, Fanton L, Cameriere R. Third molar maturity index for indicating the legal adult age in southeastern France. Forensic science international 2019;294:218.e211-218.e216. | Does not match PIRO |
|  | Tan Y, Wang J, Ba K, Zhang S, Chen J, Luo Z, et al. Relationship between dental calcification stages of the third molar and ages among teenagers in Chengdu. Hua xi kou qiang yi xue za zhi = Huaxi kouqiang yixue zazhi = West China journal of stomatology 2013;31:272-278. | Language |
|  | Tangmose S, Thevissen P, Lynnerup N, Willems G, Boldsen J. Age estimation in the living: Transition analysis on developing third molars. Forensic science international 2015;257:512.e511-512.e517. | Does not match PIRO |
|  | Tao J, Wang Y, Liu Rj, Xu X, Li Xp. Accuracy of age estimation from orthopantomograph using Demirjian's method. Fa yi xue za zhi 2007;23:258-260. | Language |
|  | Thevissen PW, Fieuws S, Willems G. Human dental age estimation using third molar developmental stages: does a Bayesian approach outperform regression models to discriminate between juveniles and adults? International journal of legal medicine 2010;124:35-42. | Does not match PIRO |
|  | Thevissen PW, Galiti D, Willems G. Human dental age estimation combining third molar(s) development and tooth morphological age predictors. International journal of legal medicine 2012;126:883-887. | Does not match PIRO |
|  | Thevissen PW, Fieuws S, Willems G. Third molar development: evaluation of nine tooth development registration techniques for age estimations. Journal of forensic sciences 2013;58:393-397. | Does not match PIRO |
|  | Thorson J, Hagg U. The accuracy and precision of the third mandibular molar as an indicator of chronological age. Swedish dental journal 1991;15:15-22. | Does not match PIRO |
|  | Toth ZO, Udvar O, Angyal J. Chronological age estimation based on dental panoramic radiography. Kormeghatarozas panorama rontgenfelvetelek alapjan. 2014;107:93-98. | Language |
|  | Trakiniene G, Smailiene D, Kuciauskiene A. Evaluation of skeletal maturity using maxillary canine, mandibular second and third molar calcification stages. European journal of orthodontics 2016;38:398-403. | Does not match PIRO |
|  | Trevino-Tijerina MC, Valenzuela-Garach A, Elizondo-Pereo RA, Cerda-Flores RM, Vargas-Villarreal J, González-Salazar F. Age estimation of teenagers from Monterrey (Mexico) by the evaluation of dental mineralization after multi-slice helical computed tomography. Australian Journal of Forensic Sciences 2016;48:138-149. | Does not match PIRO |
|  | Tuteja M, Bahirwani S, Balaji P. An evaluation of third molar eruption for assessment of chronologic age: A panoramic study. Journal of forensic dental sciences 2012;4:13-18. | Does not match PIRO |
|  | Uys A, Fabris-Rotelli I, Bernitz H. Estimating age in black South African children. SADJ : journal of the South African Dental Association = tydskrif van die Suid-Afrikaanse Tandheelkundige Vereniging 2014;69:54-51. | Does not match PIRO |
|  | Uzuner FD, Kaygisiz E, Yeniay A, Darendeliler N, Zor ZF. Radiographic evaluation of third molar development in relation to chronological age, gender and jaws. Journal of Oral and Maxillofacial Surgery 2014;72:e80-e81. | Does not match PIRO |
|  | Van Vlierberghe M, Boltacz-Rzepkowska E, Van Langenhove L, Laszkiewicz J, Wyns B, Devlaminck D, et al. A comparative study of two different regression methods for radiographs in Polish youngsters estimating chronological age on third molars. Forensic science international 2010;201:86-94. | Does not match PIRO |
|  | Verochana K, Prapayasatok S, Janhom A, Mahasantipiya PM, Korwanich N. Accuracy of an equation for estimating age from mandibular third molar development in a Thai population. Imaging science in dentistry 2016;46:1-7. | Does not match PIRO |
|  | Wang J, Bai X, Wang M, Zhou Z, Bian X, Qiu C, et al. Applicability and accuracy of Demirjian and Willems methods in a population of Eastern Chinese subadults. Forensic science international 2018;292:90-96. | Does not match PIRO |
|  | Wang J, Ji F, Zhai Y, Park H, Tao J. Is Willems method universal for age estimation: a systematic review and meta-analysis. J Forensic Leg Med 2017; 52:130-6. | Does not match PIRO |
|  | Wang J, Wang M, Shen S, Guo Y, Fan L, Ji F, et al. Testing the nonlinear equations for dental age evaluation in a population of eastern China. Legal medicine (Tokyo, Japan) 2021;48:101793. | Does not match PIRO |
|  | Wang M, Fan L, Shen S, Bai X, Wang J, Ji F, et al. Applicability of the third molar maturity index for assessment of age of majority in Eastern China. Legal medicine (Tokyo, Japan) 2019;41:101639. | Does not match PIRO |
|  | Wang M, Wang J, Pan Y, Fan L, Shen Z, Ji F, et al. Applicability of newly derived second and third molar maturity indices for indicating the legal age of 16 years in the Southern Chinese population. Legal medicine (Tokyo, Japan) 2020;46:101725. | Does not match PIRO |
|  | Wang MTA, Huang B, Chiu AHY, Lam WM, Takahashi K, Bessho K, et al. Discrepancy between chronological age and evaluated dental age using the Demirjian system in Western Australian children. Australian Journal of Forensic Sciences 2015;47:469-474. | Does not match PIRO |
|  | Widek T, Genet P, Merkens H, Boldt J, Petrovic A, Vallis J, et al. Dental age estimation: The chronology of mineralization and eruption of male third molars with 3T MRI. Forensic science international 2019;297:228-235. | Does not match PIRO |
|  | Willems G, Van Olmen A, Spiessens B, Carels C. Dental age estimation in Belgian children: Demirjian's technique revisited. Journal of forensic sciences 2001;46:893-895. | Does not match PIRO |
|  | Willems G, Thevissen PW, Belmans A, Liversidge HM. Willems II. Non-gender-specific dental maturity scores. Forensic science international 2010;201:84-85. | Does not match PIRO |
|  | Wolf TG, Briseno-Marroquin B, Callaway A, Patyna M, Muller VT, Willershausen I, et al. Dental age assessment in 6- to 14-year old German children: comparison of Cameriere and Demirjian methods. BMC oral health 2016;16:120. | Does not match PIRO |
|  | Wong HM, Wen YF, Jayaraman J, Li J, Sun L, King NM, et al. Northern Chinese dental ages estimated from southern Chinese reference datasets closely correlate with chronological age. Heliyon 2016;2:e00216. | Does not match PIRO |
|  | Yan J, Lou X, Xie L, Yu D, Shen G, Wang Y. Assessment of dental age of children aged 3.5 to 16.9 years using Demirjian's method: a meta-analysis based on 26 studies. PloS one 2013;8:e84672. | Does not match PIRO |
|  | Yang Z, Geng K, Liu Y, Sun S, Wen D, Xiao J, et al. Accuracy of the Demirjian and Willems methods of dental age estimation for children from central southern China. International journal of legal medicine 2019;133:593-601. | Does not match PIRO |
|  | Yusof MYPM, Thevissen PW, Fieuws S, Willems G. Dental age estimation in Malay children based on all permanent teeth types. International journal of legal medicine 2014;128:329-333. | Does not match PIRO |
|  | Zatylna N, Rogowska K, Kozanecka A. Comparison of 6-12-year-old girls' and boys' dental age using Demirijan's method. Dental and Medical Problems 2013;50:64-70. | Does not match PIRO |
|  | Zelic K, Galic I, Nedeljkovic N, Jakovljevic A, Milosevic O, Djuric M, et al. Accuracy of Cameriere's third molar maturity index in assessing legal adulthood on Serbian population. Forensic science international 2016;259:127-132. | Does not match PIRO |
|  | Zeng DL, Wu ZL, Cui MY. Chronological age estimation of third molar mineralization of Han in southern China. International journal of legal medicine 2010;124:119-123. | Does not match PIRO |
|  | Zhai Y, Park H, Han J, Wang H, Ji F, Tao J. Dental age assessment in a northern Chinese population. Journal of forensic and legal medicine 2016;38:43-49. | Does not match PIRO |
|  | Zirk M, Zoeller JE, Lentzen MP, Bergeest L, Buller J, Zinser M. Comparison of two established 2D staging techniques to their appliance in 3D cone beam computer-tomography for dental age estimation. Sci Rep.11(1):9024. | Does not match PIRO |
