# Supplementary material for: How old are you? A systematic review investigating the relationship between age and mandibular third molar maturity
Source: PLoS One. 2023 May 18;18(5):e0285252. doi: 10.1371/journal.pone.0285252 (PMC10194975; doi:10.1371/journal.pone.0285252)
Supplement: S3 Table — (DOCX) [file pone.0285252.s003.docx]

**Domain 1. PATIENT SELECTION**

**A. Risk for bias**

Describe methods of patient selection:

| **Item** | **Yes** | **Unclear** | **No** |
| --- | --- | --- | --- |
| Was the selection process well described? |  |  |  |
| Was a consecutive or random sample of patients enrolled? |  |  |  |
| Did the study avoid inappropriate exclusions/inclusion? |  |  |  |
| Even age distribution? |  |  |  |
| **Risk of bias** | low | moderate | high |
| **Could the conduct of interpretation of the index test have introduced bias?** |  |  |  |

**Domain 2. INDEX TEST**

Describe the index test and how it was conducted and interpreted:

**A. Risk for bias**

| **Item** | **Yes** | **Unclear** | **No** |
| --- | --- | --- | --- |
| Were the index test results interpreted without knowledge of the results of the reference standard? |  |  |  |
| Were the stages for molar development well described/assessed? |  |  |  |
| Was the index test assessed by more than one observer? |  |  |  |
| If both 38 and 48 are included, is the data for each tooth shown separately. (If only data for one tooth is shown, is it the tooth that is the least developed?) |  |  |  |
| **Risk of bias** | low | moderate | high |
| **Could the conduct of interpretation of the index test have introduced bias?** |  |  |  |

**Domain 3. REFERENCE STANDARD**

Describe the reference standard:

**A. Risk of bias**

| **Item** | **Yes** | **Unclear** | **No** |
| --- | --- | --- | --- |
| Is the reference standard likely to correctly classify the target condition? |  |  |  |
| **Risk of bias** | low | moderate | high |
| **Could the reference standard, its conduct or interpretation have introduced bias?** |  |  |  |

**Domain 4. FLOW AND TIMING**

**A. Risk for bias**

| **Item** | **Yes** | **Unclear** | **No** |
| --- | --- | --- | --- |
| Were all patients included in the analysis? |  |  |  |
| Were all teeth included in the analysis? |  |  |  |
| **Risk of bias** | low | moderate | high |
| **Could the patient flow have introduced bias?** |  |  |  |

**Domain 5: DATA**

**A. Relevance**

| **Item** | **Yes** | **Unclear** | **No** |
| --- | --- | --- | --- |
| Is the data presented without any issues? |  |  |  |
| Age Mimicry: even age distribution? If no, was it correctly analysed in the results? |  |  |  |
| **Risk of bias** | low | moderate | high |
| **Could the patient flow have introduced bias?** |  |  |  |
