# Supplementary material for: How old are you? A systematic review investigating the relationship between age and mandibular third molar maturity
Source: PLoS One. 2023 May 18;18(5):e0285252. doi: 10.1371/journal.pone.0285252 (PMC10194975; doi:10.1371/journal.pone.0285252)
Supplement: S4 Table — (DOCX) [file pone.0285252.s004.docx]

**S3. Quality assessment of studies with high risk of bias**

|  | Patient selection | Index  test | Reference standard | Flow and timing | Data |
| --- | --- | --- | --- | --- | --- |
| Acharya AB, 2011 |  |  |  |  |  |
| Ajmal M et al., 2012 |  |  |  |  |  |
| Al-Balbeesi HO et al., 2018 |  |  |  |  |  |
| Alshihri AM et al., 2014 |  |  |  |  |  |
| Amanullah A et al., 2016 |  |  |  |  |  |
| Arany S et al., 2004 |  |  |  |  |  |
| Ashifa N et al., 2020 |  |  |  |  |  |
| Babburi S et al., 2015 |  |  |  |  |  |
| Berkvens ME et al., 2017 |  |  |  |  |  |
| Cameriere R et al., 2008 |  |  |  |  |  |
| Cavric J et al., 2016 |  |  |  |  |  |
| Costa J et al., 2014 |  |  |  |  |  |
| De Salvia A et al., 2004 |  |  |  |  |  |
| Elshehawi W et al., 2016 |  |  |  |  |  |
| Filipović G et al., 2020 |  |  |  |  |  |
| Friedrich RE et al., 2016 |  |  |  |  |  |
| Gaeta-Araujo H et al., 2021 |  |  |  |  |  |
| Garamendi PM et al., 2005 |  |  |  |  |  |
| Ismail M et al., 2016 |  |  |  |  |  |
| Johan NA et al., 2012 |  |  |  |  |  |
| Jung YH et al., 2014 |  |  |  |  |  |
| Karadayi B et al., 2015 |  |  |  |  |  |
| Khosronejad A et al., 2017 |  |  |  |  |  |
| Knell B et al., 2009 |  |  |  |  |  |
| Lee SH et al., 2009 |  |  |  |  |  |
| Lewis AJ et al., 2015 |  |  |  |  |  |
| Liu Y et al., 2018 |  |  |  |  |  |
| Lucas VS et al., 2016 |  |  |  |  |  |
| Maled V, Vishwanath SB, 2016 |  |  |  |  |  |
| Mehta N et al., 2016 |  |  |  |  |  |
| Mincer HH et al., 1993 |  |  |  |  |  |
| Naik SB et al., 2014 |  |  |  |  |  |
| Nur BG et al., 2015 |  |  |  |  |  |
| Olze A et al., 2003 |  |  |  |  |  |
| Olze A et al., 2004 |  |  |  |  |  |
| Olze A et al., 2010 |  |  |  |  |  |
| Orhan K et al., 2007 |  |  |  |  |  |
| Padubidri JR et al., 2018 |  |  |  |  |  |
| Pinares Toledo J et al., 2021 |  |  |  |  |  |
| Prieto JL et al., 2005 |  |  |  |  |  |
| Priyadharshini KI et al., 2015 |  |  |  |  |  |
| Qing M et al., 2014 |  |  |  |  |  |
| Rai B et al., 2010 |  |  |  |  |  |
| Ramaswami TB et al., 2020 |  |  |  |  |  |
| Rougé-Maillart C et al., 2011 |  |  |  |  |  |
| Saranaya K et al., 2021 |  |  |  |  |  |
| Solari AC, Abramovitch K, 2002 |  |  |  |  |  |
| Streckbein P et al., 2014 |  |  |  |  |  |
| Upalananda W et al., 2021 |  |  |  |  |  |
| Uys A et al., 2018 |  |  |  |  |  |

Low risk of bias in green, unclear risk of bias in yellow, high risk of bias in red.

**References, studies with high risk of bias**

1. Acharya AB. Accuracy of predicting 18 years of age from mandibular third molar development in an Indian sample using Demirjian's ten-stage criteria. International journal of legal medicine 2011;125:227-233.
2. Ajmal M, Assiri KI, Al-Ameer KY, Assiri AM, Luqman M. Age estimation using third molar teeth: A study on southern Saudi population. Journal of forensic dental sciences 2012;4:63-65.
3. Al-Balbeesi HO, Al-Nahas NW, Baidas LF, Bin Huraib SM, Alhaidari Ra, Alwadai G. Correlation between skeletal maturation and developmental stages of canines and third molars among Saudi subjects. The Saudi dental journal 2018;30:74-84.
4. Alshihri AM, Kruger E, Tennant M. Western Saudi adolescent age estimation utilising third molar development. European journal of dentistry 2014;8:296-301.
5. Amanullah A, Ullah U, Yunus S, Munim A. Development stages of third-molar tooth for estimation of chronological age in children and young adult. Pakistan Journal of Medical and Health Sciences 2016;10:750-754.
6. Arany S, Iino M, Yoshioka N. Radiographic survey of third molar development in relation to chronological age among Japanese juveniles. Journal of forensic sciences. 2004;49(3):534-8.
7. Ashifa N, Parakh MK, Ulaganambi S. Estimation of Age Using Third Molar Development: A Radiological Cross-Sectional Study. The American journal of forensic medicine and pathology 2020;41:115-118.
8. Babburi S, Nelakurthi H, Aparna V, Soujanya P, Kotti AB, Ganipineni K. Radiographic Estimation of Chronological Age using Mineralization of Third Molars in Coastal Andhra, India. Journal of international oral health : JIOH 2015;7:49-52.
9. Berkvens ME, Fairgrieve SI, Keenan S. A comparison of techniques in age estimation using the third molar. Journal of the Canadian Society of Forensic Science 2017;50:74-83.
10. Cameriere R, Ferrante L, De Angelis D, Scarpino F, Galli F. The comparison between measurement of open apices of third molars and Demirjian stages to test chronological age of over 18-year-olds in living subjects. International journal of legal medicine 2008;122:493-497.
11. Cavric J, Vodanovic M, Marusic A, Galic I. Time of mineralization of permanent teeth in children and adolescents in Gaborone, Botswana. Annals of anatomy = Anatomischer Anzeiger : official organ of the Anatomische Gesellschaft 2016;203:24-32.
12. Costa J, Montero J, Serrano S, Albaladejo A, Lopez-Valverde A, Bica I. Accuracy in the legal age estimation according to the third molars mineralization among Mexicans and Columbians. Atencion primaria 2014;46:165-175.
13. De Salvia A, Calzetta C, Orrico M, De Leo D. Third mandibular molar radiological development as an indicator of chronological age in a European population. Forensic science international 2004;146:S9-S12.
14. Elshehawi W, Alsaffar H, Roberts G, Lucas V, McDonald F, Camilleri S. Dental age assessment of Maltese children and adolescents. Development of a reference dataset and comparison with a United Kingdom Caucasian reference dataset. Journal of forensic and legal medicine. 2016;39:27-33.
15. Filipović G, Djordjević NS, Stojanović NM, Brkić Z, Igić M, Marjanović D, et al. Evaluation of chronological age based on third-molar development in the Serbian population. Vojnosanitetski Pregled 2020;77:1054-1059.
16. Friedrich RE, Schmidt K, Treszl A, Kersten JF. Predictive values derived from lower wisdom teeth developmental stages on orthopantomograms to calculate the chronological age in adolescence and young adults as a prerequisite to obtain age-adjusted informed patient consent prior to elective surgical procedures in young patients with incomplete or mismatched personal data. GMS Interdisciplinary plastic and reconstructive surgery DGPW 2016;5:Doc23.
17. Gaeta-Araujo H, Oliveira-Santos N, Nascimento EHL, Nogueira-Reis F, Oenning AC, Groppo FC, et al. A new model of classification of third molars development and its correlation with chronological age in a Brazilian subpopulation. International journal of legal medicine 2021;135:639-648.
18. Garamendi PM, Landa MI, Ballesteros J, Solano MA. Reliability of the methods applied to assess age minority in living subjects around 18 years old. A survey on a Moroccan origin population. Forensic science international 2005;154:3-12.
19. Ismail M, Venkata Naga Mohan Rao B, Mohinuddin K. A study of age estimation using third molar teeth in and around Warangal area. Medico-Legal Update 2016;16:12-15.
20. Johan NA, Khamis MF, Abdul Jamal NS, Ahmad B, Mahanani ES. The variability of lower third molar development in Northeast Malaysian population with application to age estimation. The Journal of forensic odonto-stomatology 2012;30:45-54.
21. Jung YH, Cho BH. Radiographic evaluation of third molar development in 6- to 24-year-olds. Imaging science in dentistry 2014;44:185-191.
22. Karadayi B, Kaya A, Afsin H, Ozaslan A, Çetin G. The usage of third molars to determine legally relevant age thresholds in Turkey. Australian Journal of Forensic Sciences 2015;47:275-282.
23. Khosronejad A, Navabi M, Sakhdari S, Rakhshan V. Correlation between chronological age and third molar developmental stages in an Iranian population (Demirjian method). Dental research journal 2017;14:143-149.
24. Knell B, Ruhstaller P, Prieels F, Schmeling A. Dental age diagnostics by means of radiographical evaluation of the growth stages of lower wisdom teeth. International journal of legal medicine 2009;123:465-469.
25. Lee SH, Lee J Y, Park H K, Kim Y K. Development of third molars in Korean juveniles and adolescents. Forensic science international- 2009; 188: 107-111.
26. Lewis AJ, Boaz K, Nagesh KR, Srikant N, Gupta N, Nandita KP, et al. Demirjian's method in the estimation of age: A study on human third molars. Journal of forensic dental sciences 2015;7:153-157.
27. Liu Y, Geng K, Chu Y, Xu M, Zha L. Third molar mineralization in relation to chronologic age estimation of the Han in central southern China. International journal of legal medicine. 2018;132(5):1427-35.
28. Lucas VS, Andiappan M, McDonald F, Roberts G. Dental Age Estimation: A Test of the Reliability of Correctly Identifying a Subject Over 18 Years of Age Using the Gold Standard of Chronological Age as the Comparator. Journal of forensic sciences 2016;61:1238-1243.
29. Maled V, Vishwanath SB. The chronology of third molar mineralization by digital orthopantomography. Journal of forensic and legal medicine 2016;43:70-75.
30. Mehta N, Patel D, Mehta F, Gupta B, Zaveri G, Shah U. Evaluation of skeletal maturation using mandibular third molar development in Indian adolescents. Journal of forensic dental sciences 2016;8:112.
31. Mincer HH, Harris EF, Berryman HE. The A.B.F.O. study of third molar development and its use as an estimator of chronological age. Journal of forensic sciences 1993;38:379-390.
32. Naik SB, Patil SN, Kamble SD, Mowade T, Motghare P. Reliability of Third Molar Development for Age Estimation by Radiographic Examination (Demirjian's Method). Journal of clinical and diagnostic research : JCDR 2014;8:ZC25-28.
33. Nur BG, Altunsoy M, Akkemik O, Ok E, Evcil MS. Third-molar mineralization and eruption correlated to chronologic age in Turkish children and adolescents. Australian Journal of Forensic Sciences 2015;47:313-321.
34. Olze A, Taniguchi M, Schmeling A, Zhu BL, Yamada Y, Maeda H, et al. Comparative study on the chronology of third molar mineralization in a Japanese and a German population. Legal medicine (Tokyo, Japan) 2003;5:S256-260.
35. Olze A, Taniguchi M, Schmeling A, Zhu BL, Yamada Y, Maeda H, et al. Studies on the chronology of third molar mineralization in a Japanese population. Legal medicine (Tokyo, Japan) 2004;6:73-79.
36. Olze A, Pynn BR, Kraul V, Schulz R, Heinecke A, Pfeiffer H, Schmeling, A. Studies on the chronology of third molar mineralization in First Nations people of Canada. International journal of legal medicine 2010; 124: 433-437.
37. Orhan K, Ozer L, Orhan AI, Dogan S, Paksoy CS. Radiographic evaluation of third molar development in relation to chronological age among Turkish children and youth. Forensic science international 2007;165:46-51.
38. Padubidri JR, Kongara S, Rao SJ, Udupa R, Kotian MS. Radiological development of mandibular third molar as an indicator of chronological age itn South Indian population. Medico-Legal Update 2018;18:52-58.
39. Pinares Toledo J, Retamal Yermani R, Ortega Pinto A, Villanueva Conejeros R. Development of the third molar in Chileans: A radiographic study on chronological age. Forensic Science International: Reports 2021;3.
40. Prieto JL, Barberia E, Ortega R, Magana C. Evaluation of chronological age based on third molar development in the Spanish population. International journal of legal medicine 2005;119:349-354.
41. Priyadharshini KI, Idiculla JJ, Sivapathasundaram B, Mohanbabu V, Augustine D, Patil S. Age estimation using development of third molars in South Indian population: A radiological study. Journal of International Society of Preventive & Community Dentistry 2015;5:S32-38.
42. Qing M, Qiu L, Gao Z, Bhandari K. The chronological age estimation of third molar mineralization of Han population in southwestern China. Journal of forensic and legal medicine. 2014;24:24-7.
43. Rai B, Kaur J, Jafarzadeh H. Dental age estimation from the developmental stage of the third molars in Iranian population. Journal of forensic and legal medicine 2010;17:309-311.
44. Ramaswami TB, Rosa GCD, Fernandes MM, Oliveira RND, Tinoco RLR. Third molar development by Demirjian's stages and age estimation among Brazilians. Forensic Imaging 2020;20.
45. Rougé-Maillart C, Franco A, Franco T, Jousset N. Estimation of the age of 15-25 year-olds using Dermirjian's dental technique. Study of a population from the West, France. Revue de Medecine Legale. 2011;2(3):117-24.
46. Saranya K, Ponnada SR, Cheruvathoor JJ, Jacob S, ukuri G, Mudigonda M, et al. Assessing the probability of having attained 16 years of age in juveniles using third molar development in a sample of South Indian population. J Forensic Odontostomatol.1(39):16-23.
47. Solari AC, Abramovitch K. The accuracy and precision of third molar development as an indicator of chronological age in Hispanics. Journal of forensic sciences 2002;47:531-535.
48. Streckbein P, Reichert I, Verhoff MA, Bodeker RH, Kahling C, Wilbrand JF, et al. Estimation of legal age using calcification stages of third molars in living individuals. Science & justice : journal of the Forensic Science Society 2014;54:447-450.
49. Upalananda W, Wantanajittikul K, Na Lampang S, Janhom A. Semi-automated technique to assess the developmental stage of mandibular third molars for age estimation. Australian Journal of Forensic Sciences 2021.
50. Uys A, Bernitz H, Pretorius S, Steyn M. Estimating age and the probability of being at least 18 years of age using third molars: a comparison between Black and White individuals living in South Africa. International journal of legal medicine. 2018;132(5):1437-46.
